# Bifunctional recyclable ZnO/MgO nanocomposite: solvent-free synthesis of chromenes and efficient water remediation

**DOI:** 10.1038/s41598-026-43572-y

**Published:** 2026-05-07

**Authors:** Wael A. A. Arafa, AbdElAziz A. Nayl, Ahmed H. Alanazi, Ismail M. Ahmed, Adel A. Abdelwahab, Hamada Mohamed Ibrahim, Stefan Bräse, Amr Mohammad Nassar

**Affiliations:** 1 Department of Chemistry, College of Science, Jouf University, 72341 Al Jouf, Sakaka Saudi Arabia; 2 Department of Chemistry, Faculty of Science, Fayoum University, Fayoum, 63514 Egypt; 3 Institute of Biological and Chemical Systems-Functional Molecular Systems (IBCS-FMS), Kaiserstrasse 12, 76131 Karlsruhe, Germany

**Keywords:** Chromenes, ZnO/MgO nanocomposite, Solvent-free synthesis, Photocatalytic degradation, Nanostructured composite, Green chemistry, Water remediation, Chemistry, Environmental sciences, Materials science, Nanoscience and technology

## Abstract

A ZnO/MgO nanostructured composite was prepared through the thermal degradation of a mixed zinc oxalate/magnesium oxalate precursor. The obtained ZnO/MgO has been characterized using thermal gravimetric analysis (TGA), scanning electron microscopy (SEM), X-ray diffraction (XRD), and Fourier transform infrared spectroscopy (FTIR). The XRD reveals a crystalline structure with a crystal size of 33 nm. The TGA confirmed the purity and stability of ZnO/MgO NPs at high temperatures. The ZnO/MgO NPs served as an efficient heterogeneous catalyst for the solvent-free, room-temperature Knoevenagel-Michael domino assembly of a library of 2-amino-4*H*-chromenes. The designed catalyst offers short reaction times (8–12 min), excellent yields (91–99%), easy recovery and reuse for at least 4 cycles without a noticeable drop in efficacy, and maintains good performance in scale-up experiments. Furthermore, the composite exhibited promising photocatalytic activity, achieving 96.36% removal of methyl orange dye (5.0 ppm) under solar irradiation within 30 min, outperforming pure MgO (3.64%) and ZnO (18.18%). Comprehensive post-reaction characterization reveals that ZnO/MgO retains its structural and chemical integrity following both heterogeneous organic catalysis and photocatalytic dye degradation. These findings indicate that ZnO/MgO exhibits promising catalytic and photocatalytic performance under the investigated conditions.

**Supplementary Information:**

The online version contains supplementary material available at 10.1038/s41598-026-43572-y.

## Introduction

**Fig. 1:**
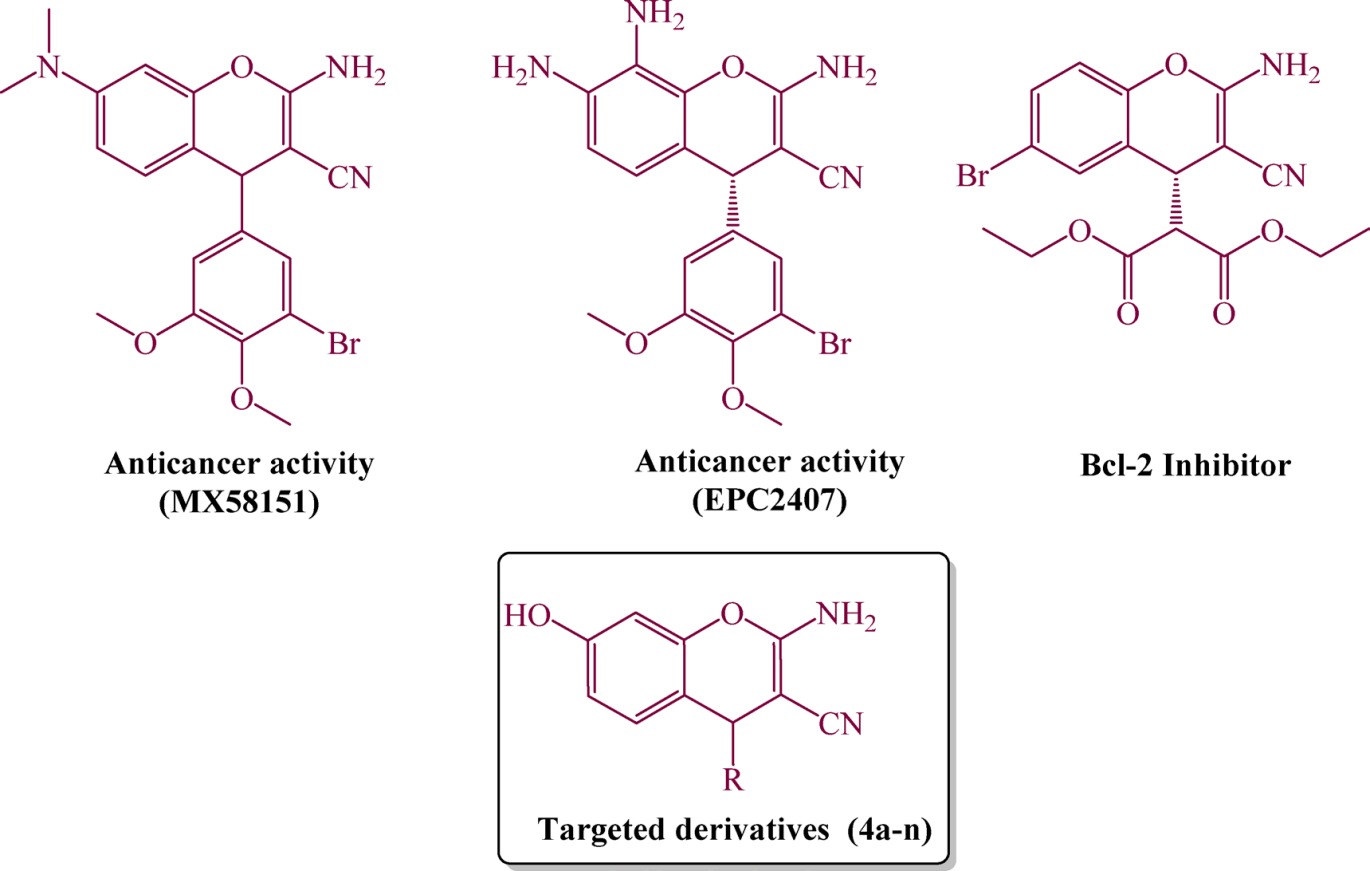
Representative chromene-containing compounds with medicinal applications and the corresponding targeted derivatives (**4a–n**).

Mixed oxides act as an essential part in the chemistry and applications of inorganic composites^1^. The vital role of mixed oxides stems from their applications in several environmental^2^, industrial^3^, biological^4^, and medical^5^ fields. ZnO/MgO is a distinct mixed-metal oxide because it exhibits high surface reactivity and mechanical, chemical, and biocompatible properties^6^. ZnO/MgO showed multifunctionality such as antibacterial^7^, antifungal activity, and photocatalytic properties in methylene blue removal^8^, biosensor^9^, and in solar cells^10^. One hazardous anionic azo dye with low biodegradability is methyl orange (MO)^11^. Chemistry labs, printing paper, food, textile water, and the pharmaceutical industry all use it. Because of its toxicity, it should be removed from wastewater, as it poses a risk to human health and the aquatic ecosystem^12^. Chromenes are considered as a noteworthy class of organic substances due to their broad range and versatility of biological activities^13^. Plenty of bioactive substances comprise these skeletal motifs, especially in the domains of pharmacology and therapeutic chemistry^14^ (Fig. 1). Their diverse biological activities, along with their structural flexibility, have made chromenes a key focus for organic chemists, particularly those interested in drug discovery and the development of new synthetic methods. Chromenes’ medicinal diversity, which includes anticancer, antiviral, antimicrobial, antioxidant, anti-inflammatory, and neuroprotective effects, suggests they are promising drug discovery candidates^14,15^. Furthermore, chromenes have shown substantial interest for their potential in the therapy of neurodegenerative diseases such as Alzheimer’s disease and schizophrenia^16,17^. In addition, chromenes have found several applications beyond their role in pharmacology, including biodegradable agrochemicals, cosmetic agents, and food additives, attracting the interest of several chemists over the last decade^18^. Lately, multicomponent reactions (MCRs) have received growing consideration for the preparation of organic substances, among them chromenes and their derivatives^19–21^. These reactions are remarkably efficient; they permit the simultaneous creation of multiple bonds in a single step, consequently speeding up synthetic procedures and limiting by-products, rendering them more sustainable and cost-effective^21^. While every reported protocol offers many advantages, some catalysts and reaction protocols also pose challenges and limitations. Some reported strategies may have several drawbacks, particularly the utilization of high-cost catalysts, time-consuming processes, poor reaction yields, the need for harsh conditions, and the generation of environmental wastes. Consequently, the present study focuses on the green preparation and characterization of ZnO/MgO NPs as a heterogeneous catalyst for Knoevenagel–Michael chromene synthesis. In addition, the catalyst was evaluated for the photocatalytic degradation of MO. This dual functionality highlights its applicability in chemical and environmentally relevant applications and supports its suitability for future scale-up.

## Experimental section

### Instrumentation and materials

A SHIMADZU TGA-51 was used for thermal gravimetric analysis (TGA). Scanning electron microscope (SEM) images were obtained using a Zeiss 1530 SEM. X-ray diffractions (XRD) were detected *via* XRD-7000 SHIMADZU with Kα copper radiation (1.5418 Å). A UV-Vis spectrophotometer (Cary 60 UV-Vis Spectrophotometer) was used to measure the absorption spectra. Fourier-Transform Infrared (FTIR) spectral data were measured on a Shimadzu IR-Tracer 100 spectrophotometer (Shimadzu Corporation, Kyoto, Japan). NMR spectra were acquired on a JEOL 600 MHz spectrometer (JEOL, Ltd., Peabody, MA, USA) utilizing DMSO-*d6* as the solvent, with chemical shifts (*δ*) reported in ppm relative to tetramethylsilane (TMS) as an internal standard. Melting points were determined in open capillary tubes on an Electrothermal apparatus (temperature range 25–400 °C) and are uncorrected. All solvents and fine chemicals were purchased from Sigma-Aldrich (St. Louis, MO, USA) and used without further purification.

### Synthesis of ZnO/MgO solid solution

The solid solution ZnO/MgO has been synthesized following the method described in our previous work^22^, with the calcination time adjusted in order to improve phase homogeneity and ensure complete decomposition of the oxalate precursor. Thus, 1.26 g of oxalic acid dihydrate (10.0 mmol) was added to a mixture of aqueous solutions of zinc nitrate (1.49 g, 5.0 mmol) and magnesium nitrate (0.92 g, 5.0 mmol). The mixture was stirred for 1 h at 70 °C. After that, the precipitate was filtered, washed several times with hot water, and left to dry at room temperature. The mixed oxalate sample was heated to 500 °C in a muffle for 3 h to form a ZnO/MgO solid solution.

### General protocol for the preparation of 2-amino-4*H*-chromenes 4a–n

A mixture of aldehydes (1.0 mmol), resorcinol (1.0 mmol), malononitrile (1.0 mmol), and MgO/ZnO (20.0 mg) was manually ground at room temperature using a standard laboratory mortar and pestle for 8 min. To ensure reproducibility, the grinding was performed under consistent conditions, applying comparable grinding time across all experiments. The progress of the reaction was monitored by TLC using a MeOH/DCM (1:9) solvent system, selected for its optimal separation of starting materials and products. Under these conditions, no significant side products were observed, and the reactions proceeded to complete conversion. Upon completion, the reaction mixture was extracted with dichloromethane (DCM) to remove the catalyst, and the pure products (**4a–n**) were obtained by evaporating the DCM under reduced pressure. The purity of all products was confirmed by ¹H NMR spectroscopy, melting point determination (See SI), and TLC, and all reported yields correspond to these isolated and purified products. The MgO/ZnO catalyst was separated from the reaction mixture by filtration, thoroughly washed with EtOH, dried at 80 °C under vacuum for 2 h, and reused in subsequent reactions without further activation, ensuring consistent catalytic activity across multiple cycles.

### Photocatalytic experiments

Methyl orange dye was used in this work as an organic pollutant to evaluate the photocatalytic efficiency of MgO, ZnO, and ZnO/MgO. 0.1 g of photocatalyst was mixed with 50.0 mL of 5.0 ppm methyl orange (MO) solution (pH = 7.6). The mixture was sonicated for 5 min and then exposed to natural solar irradiation of 40 × 10^3^ Lux (measured by a calibrated lux meter). Every 5 min, a 2.0 mL sample of the treated MO solution was measured using a UV-Vis spectrophotometer to determine the absorbance of the MO chromophore (λ_max_ 465 nm).

## Results and discussion

### 3.1. Characterization of ZnO/MgO Nanoparticles

#### X-ray Diffraction (XRD)

**Fig. 2:**
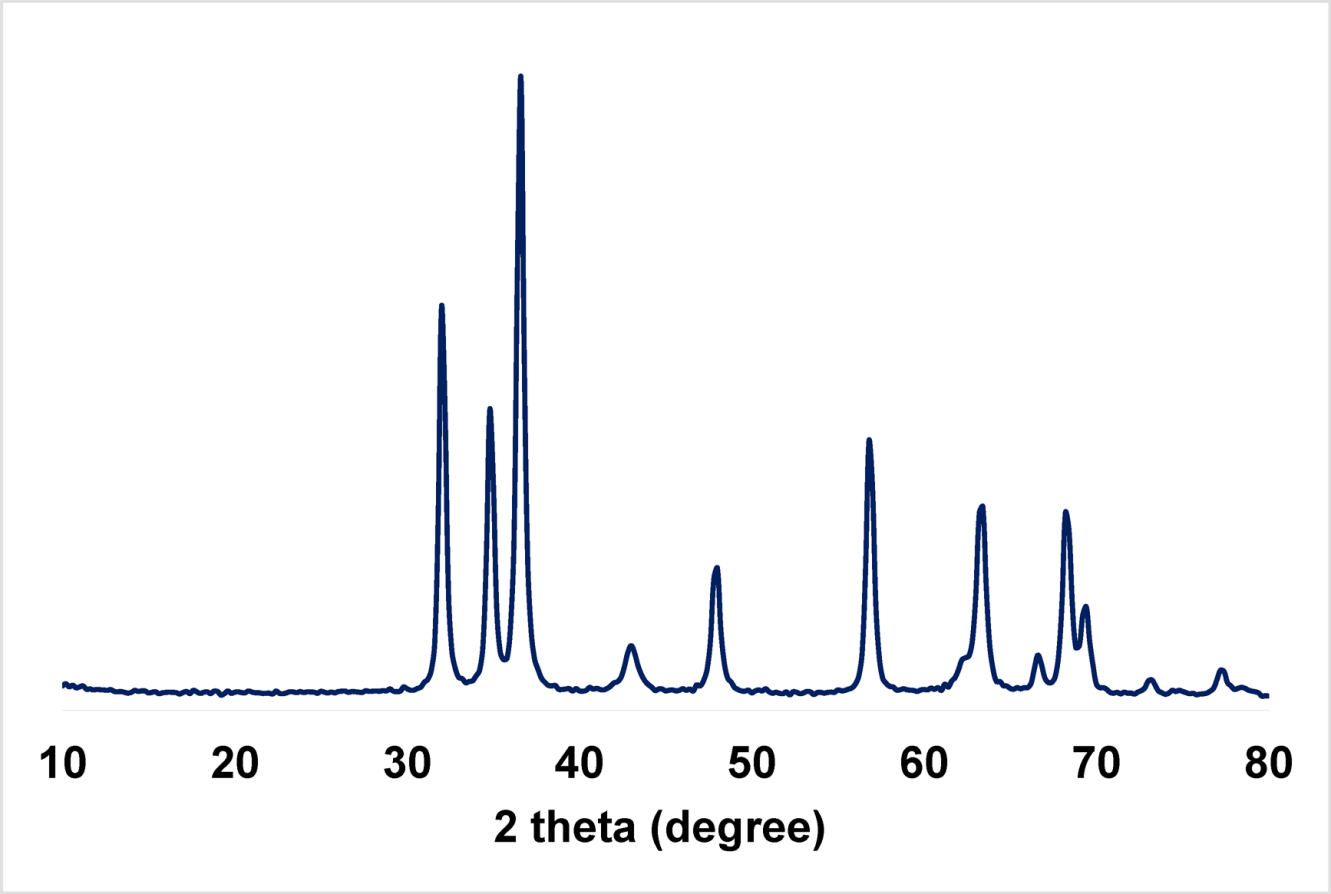
XRD of ZnO/MgO solid solution.

The XRD pattern of ZnO/MgO (Fig. 2) exhibits well-defined solid solution diffraction peaks with no separate peaks surpassing pure ZnO or MgO were detected that can be assigned to single crystalline phase formation, which indicates that a mixed oxide phase was successfully formed rather than a simple physical mixture of the two single oxides. A slight shift in the diffraction peaks compared with those of pure ZnO reflections (JCPDS No. 36-1451)^23^, and MgO reflections (JCPDS card No. 45–0946)^24^, suggests that MgO has entered the ZnO lattice. This is attributed to the ionic radii of Mg² (0.57 Å) and Zn² (0.60 Å), which allows the formation of the wurtzite structure by occupation of magnesium oxide in zinc oxide sites^25^. The peaks showed low intensity and a broad nature, which reflects the decrease in crystallinity due to the interactions between MgO and ZnO phases^26^. The average crystallite size, calculated from the Scherrer equation^27^, is 33 nm. These findings indicate the successful thermal decomposition of the mixed oxalate precursor to the formation of a single-phase MgO–ZnO solid solution.

#### Thermogravimetric analysis (TGA)

**Fig. 3:**
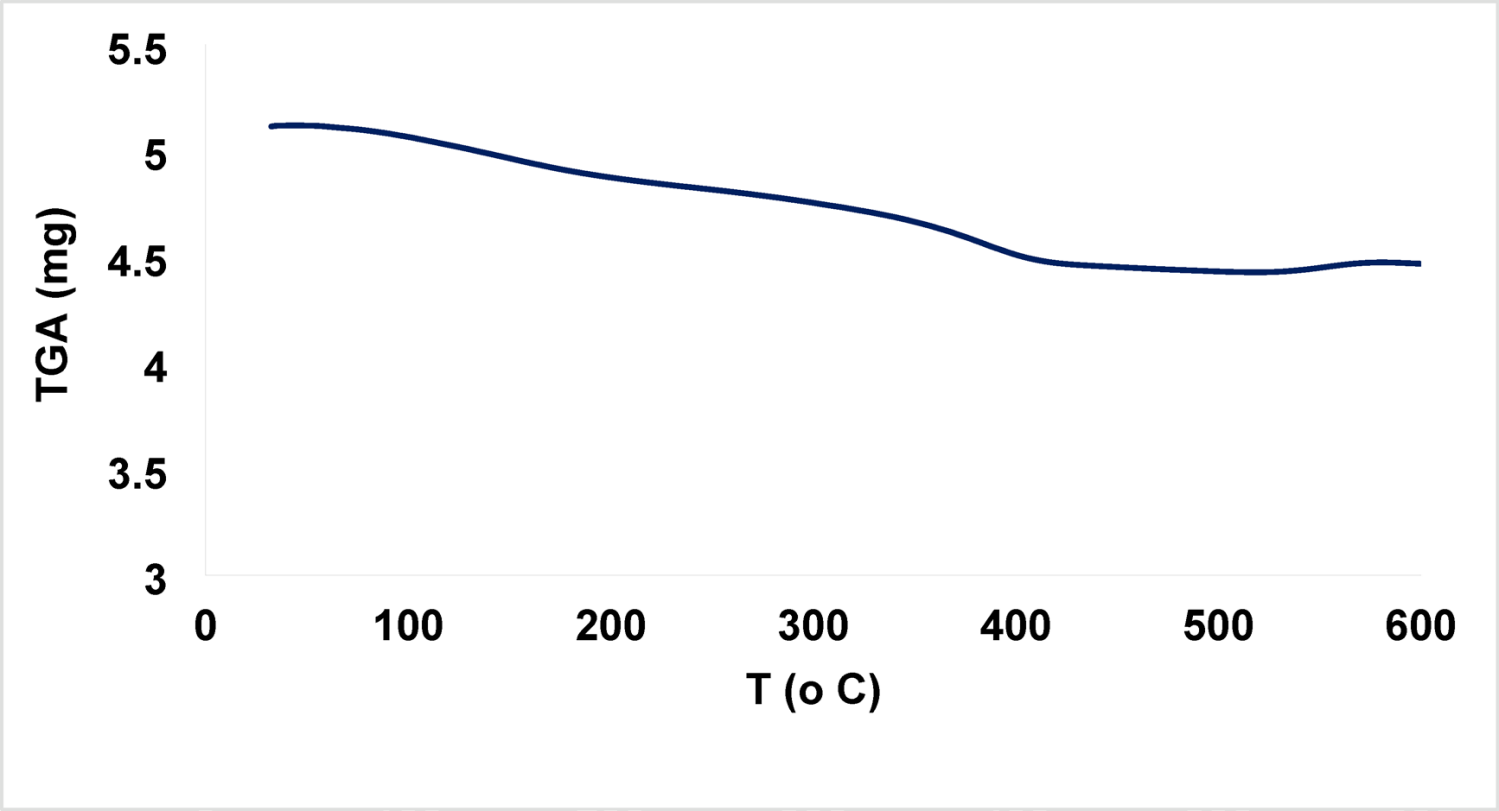
TGA of ZnO/MgO solid solution.

The TGA curve of the ZnO/MgO solid solution, Fig. 3, shows two thermal decomposition steps with a total ≈ 9.8% of weight loss. The first step, below 150 °C, with a weight loss ≈ 3.8%, corresponds to the evaporation of adsorbed water^28^. The second step is between 300 and 450 °C, with a weight loss ≈ 6%, which can be attributed to the release of adsorbed CO₂^29^. At 450 °C, the curve becomes almost flat and smooth, with no further weight change upon heating, suggesting a high thermal stability of the composite at high temperatures.

#### Scanning electron microscopy (SEM) and energy-dispersive X-ray spectroscopy (EDX)

**Fig. 4:**
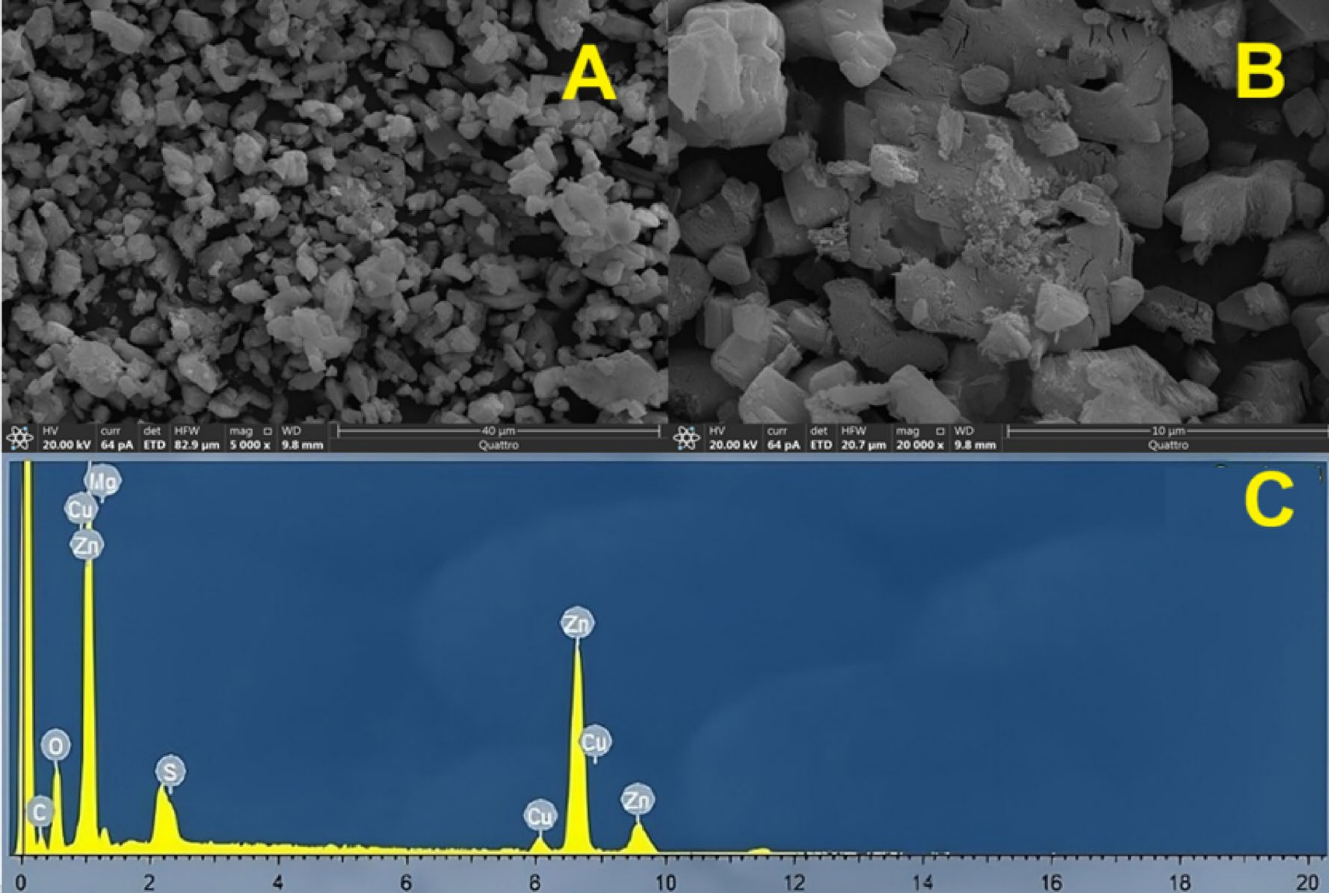
SEM micrographs at different magnifications (**A**, **B**) and corresponding EDX spectrum (**C**) of the ZnO/MgO solid solution.

The SEM image of ZnO/MgO (Fig. 4A and B) shows that the composite consists of aggregated nanoparticles with a relatively identical size distribution in agglomerated morphology. The particles are slightly irregular, and some porosity is observed, which is expected due to the evolution of CO, CO_2_, and H_2_O gases during the thermal breakdown of the mixed oxalate precursor. The micrograph shows an even distribution of crystals with no visible separation into individual MgO or ZnO crystals. In addition, the EDX analysis (Fig. 4C) confirms the presence of Zn, Mg, and O in the sample. These results provide additional evidence supporting the formation of a ZnO/MgO solid solution rather than a physical mixture of separate oxide phases. These findings matched the XRD results and confirm the formation of a single solid-solution phase.

#### Fourier transform infrared spectroscopy (FTIR)

**Fig. 5:**
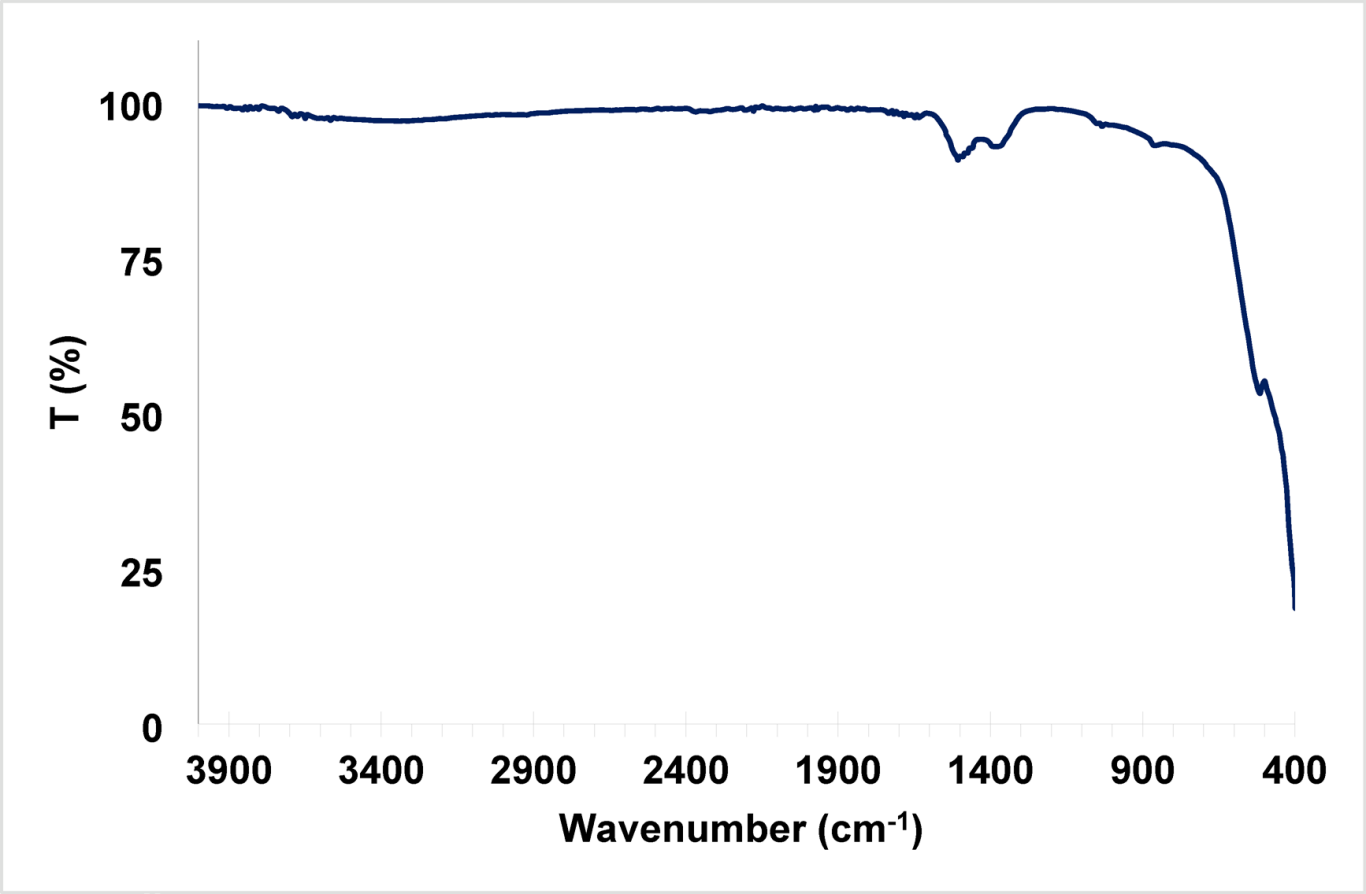
FTIR of ZnO/MgO solid solution.

The FTIR spectrum of ZnO/MgO solid solution (Fig. 5) shows a weak absorption band in the 408–609 cm^− 1^ range, which corresponds to the stretching vibrations of Mg–O and Zn–O bonds^30^. The disappearance of the distinct oxalate absorption bands between (C–O stretching) and (C–C–O bending) indicates the complete thermal decomposition of the mixed oxalate precursor. The broadened peak near 3450 cm^− 1^ arises from the stretching vibration of adsorbed water on the surface^31^. The far region exhibits the characteristic bands of ZnO–MgO at 488 and 538 cm^− 1^^32^.

### Catalytic studies for synthesis of 2‑Amino‑4*H*‑Chromenes (4a–n)

To begin with, a multicomponent reaction of benzaldehyde (**1a**, 1.0 mmol), resorcinol (**2**, 1.0 mmol), and malononitrile (**3**, 1.0 mmol) has been selected as the template reactants. The impacts of several reaction variables, namely catalyst loading, solvents, temperature, and time, have been evaluated to achieve the optimal reaction conditions (Table 1). Under solvent-free, catalyst-free conditions, the desired product (**4a**) was not observed; instead, only the starting materials and a poor yield of the Knoevenagel condensation product were detected by thin-layer chromatography (Table 1, entry 1). Likewise, the same results were observed when EtOH was used as the solvent under catalyst-free conditions (Table 1, entry 2). Next, employing 20.0 mg of the designed heterogeneous catalyst (ZnO/MgO), various solvents were assessed for the proposed reaction at both room and reflux temperatures (Table 1, entries 3–7). The results obtained indicated that polar protic solvents are more effective (Table 1, entries 3–6) than other solvents (Table 1, entry 7) in terms of yield and reaction time of the targeted chromene derivative (**4a**). Interestingly, performing the template reaction under grinding conditions (solvent-free) at ambient temperature yielded the anticipated end product (**4a**) in high yield (Table 1, entry 8). After that, the impact of the catalyst doses on the model reaction was investigated. Decreasing the catalyst dose from 20.0 mg to 15.0 mg resulted in a complex mixture with slightly reduced yield and extended reaction time (Table 1, entry 9). Moreover, increasing the catalyst dose from 20.0 to 25.0 mg did not lead to any noticeable enhancement in reaction yield or time (Table 1, entry 10). Accordingly, 20.0 mg of the catalyst was found to be the optimal dose for this conversion at ambient temperature (Table 1, entry 8). Additionally, the reaction performance was identified to be optimal in 8 min, demonstrating that this is the ideal time (Table 1, entry 11). Reducing the reaction time to less than 8 min resulted in a slightly lower yield (Table 1, entry 12), while extending the reaction time to 15 min did not lead to any improvement (Table 1, entry 13). Additionally, repeating the model reaction with MgO NPs or ZnO NPs (20.0 mg) as catalysts under the same conditions (Table 1, entries 14 & 15) led to a noticeable decrease in both yield and the rate, indicating that ZnO/MgO NPs make a significant contribution in promoting the reaction.

**Table 1:** Impact of several parameters on the synthesis of **4a**.


Entry	Solvent	Catalyst	Cat. Dose (mg)	Temp. (°C)	Time (min)	Yield (%)
1.	–	–	0	Fusion	60	0*
2.	EtOH	–	0	Reflux	60	0*
3.	EtOH	ZnO/MgO	20	Reflux	35	93
4.	EtOH	ZnO/MgO	20	rt	60	86
5.	H_2_O	ZnO/MgO	20	Reflux	30	90
6.	H_2_O	ZnO/MgO	20	rt	60	82
7.	DCM	ZnO/MgO	20	Reflux	60	35
8.	–	ZnO/MgO	20	rt	10	97
9.	–	ZnO/MgO	15	rt	15	94
10.	–	ZnO/MgO	25	rt	10	97
11.	–	ZnO/MgO	20	rt	8	97
12.	–	ZnO/MgO	20	rt	7	95
13.	–	ZnO/MgO	20	rt	15	97
14.	–	MgO	20	rt	15	91
15.	–	ZnO	20	rt	15	86

*The Knoevenagel condensation product was detected in ≈ 7%.

Under optimal conditions (Table 1, entry 11), the scope and generality of the current approach have been extended by using a variety of aldehyde derivatives (**1a–n**) in the three-component protocol, all of which are well tolerated (Table 2). The reactions proceeded smoothly, delivering the corresponding products (**4a–n**) in excellent yields (91–99%). This highlights the effectiveness and potential of the ZnO/MgO NPs catalyst in this multicomponent process. As outlined in Table 2, the ability of a reaction to work with different aldehyde types—aliphatic, aromatic, and heteroaromatic—suggests a flexible and robust catalytic system, making it more broadly applicable to a range of organic transformations. It also indicates that the steric and electronic effects of such aldehydes slightly inhibit the reaction (Table 2). Accordingly, the reactivity of aldehydes with electron-accepting motifs was found to be slightly higher than that with electron-releasing motifs. Further, *ortho*-substituents introduce steric strain, which decreases reactivity (Table 2, entries 4, 7, and 11). Thus, the position and type of substituent can therefore play a crucial role in determining the reaction outcome. The high yield (99%) for the assembly of bis-4*H*-chromene (**4n**) from *p*-phthalaldehyde (1.0 equiv), malononitrile (2.0 equiv), and resorcinol (2.0 equiv) demonstrates not only the efficiency of the protocol but also its ability to deliver complex polycyclic compounds with excellent selectivity (Table 2, entry 14). The agreement of spectroscopic results with those of known compounds in the literature not only validates the identity of the synthesized compounds (**4a–n**) but also highlights the excellent performance of the catalytic system.

**Table 2:** Nanocrystalline ZnO/MgO catalyzed three-component condensations of malononitrile, aldehydes, and resorcinol to form 2-amino-4*H*-chromenes (**4a–n**).


Entry	*R*	Product	Time (min)	Yield (%)
1.	C_6_H_5_	**4a**	8	97
2.	4-MeC_6_H_4_	**4b**	8	96
3.	4-MeOC_6_H_4_	**4c**	8	93
4.	2-MeOC_6_H_4_	**4d**	10	91
5.	4-ClC_6_H_4_	**4e**	8	97
6.	3-ClC_6_H_4_	**4f**	8	95
7.	2,4-Cl_2_C_6_H_3_	**4 g**	8	97
8.	4-BrC_6_H_4_	**4 h**	10	96
9.	4-NO_2_C_6_H_4_	**4i**	8	99
10.	3-NO_2_C_6_H_4_	**4j**	8	97
11.	2-Naphthyl	**4k**	8	94
12.	2-Furyl	**4 L**	8	97
13.	Ethyl	**4 m**	12	92
14.	OHCC_6_H_4_	**4n**	10	99

**Fig. 6:**
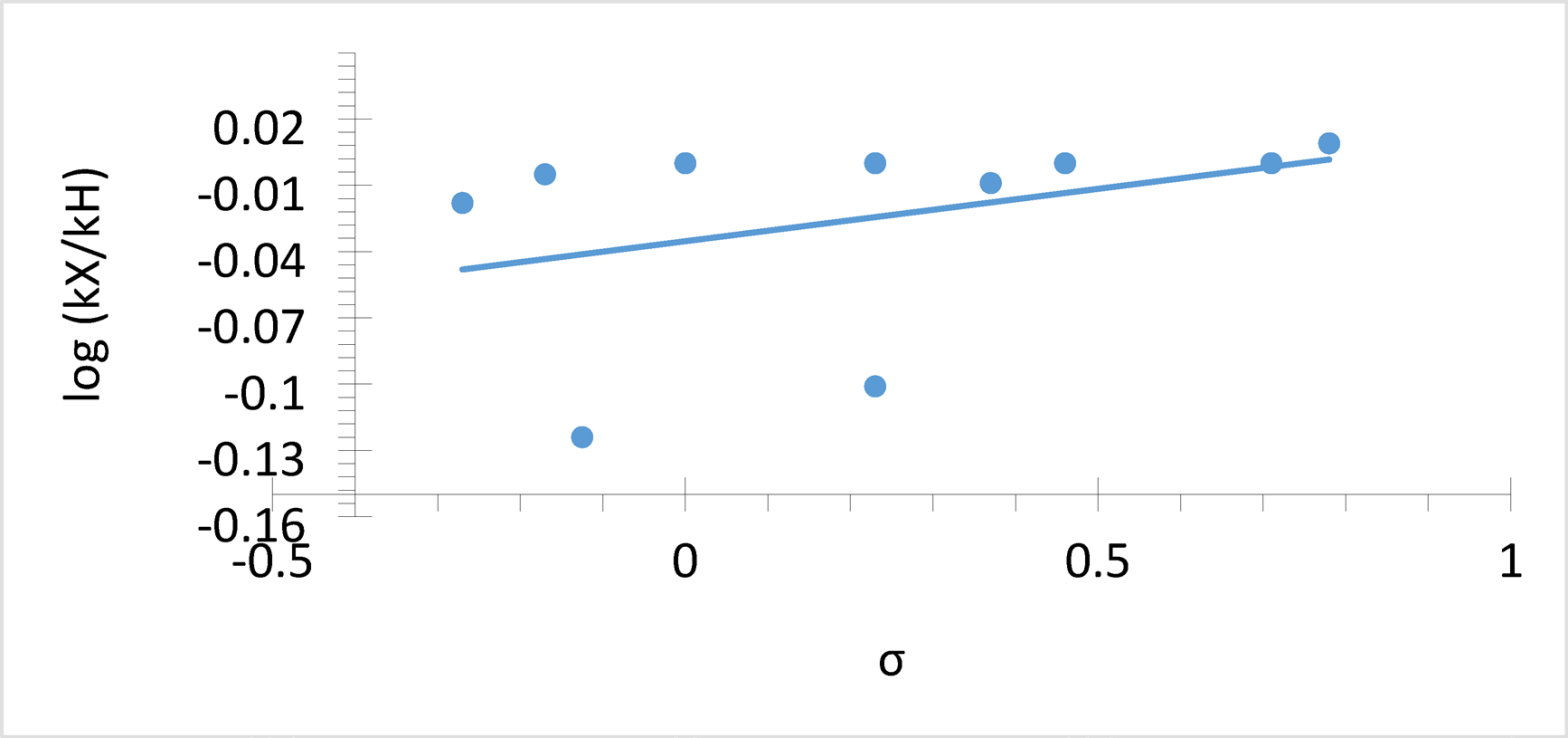
Hammett Plot of log(kX/kH) versus σ constants for substituted aromatic aldehydes in chromene formation.

To quantitatively assess the influence of substituent electronics on the reaction outcome, a Hammett-type analysis was performed using a series of meta- and para-substituted aromatic aldehydes (Fig. 6 and Table S1). The plot of log(kX/kH) versus σ constants shows a weak positive correlation (ρ = +0.015), indicating that electron-withdrawing substituents slightly accelerate the reaction. The low ρ value suggests minimal influence of aromatic substituents on the rate-determining step, disfavoring a π-activation pathway. Instead, the reaction likely proceeds *via* carbonyl activation on the ZnO/MgO surface, where Lewis acidic Zn²⁺ sites coordinate the carbonyl oxygen and adjacent basic O²⁻ sites promote nucleophilic addition and cyclization (Scheme 2). Ortho- and multiply-substituted substrates were excluded due to steric and field effects not reflected in σ constants.

**Fig. 7:**
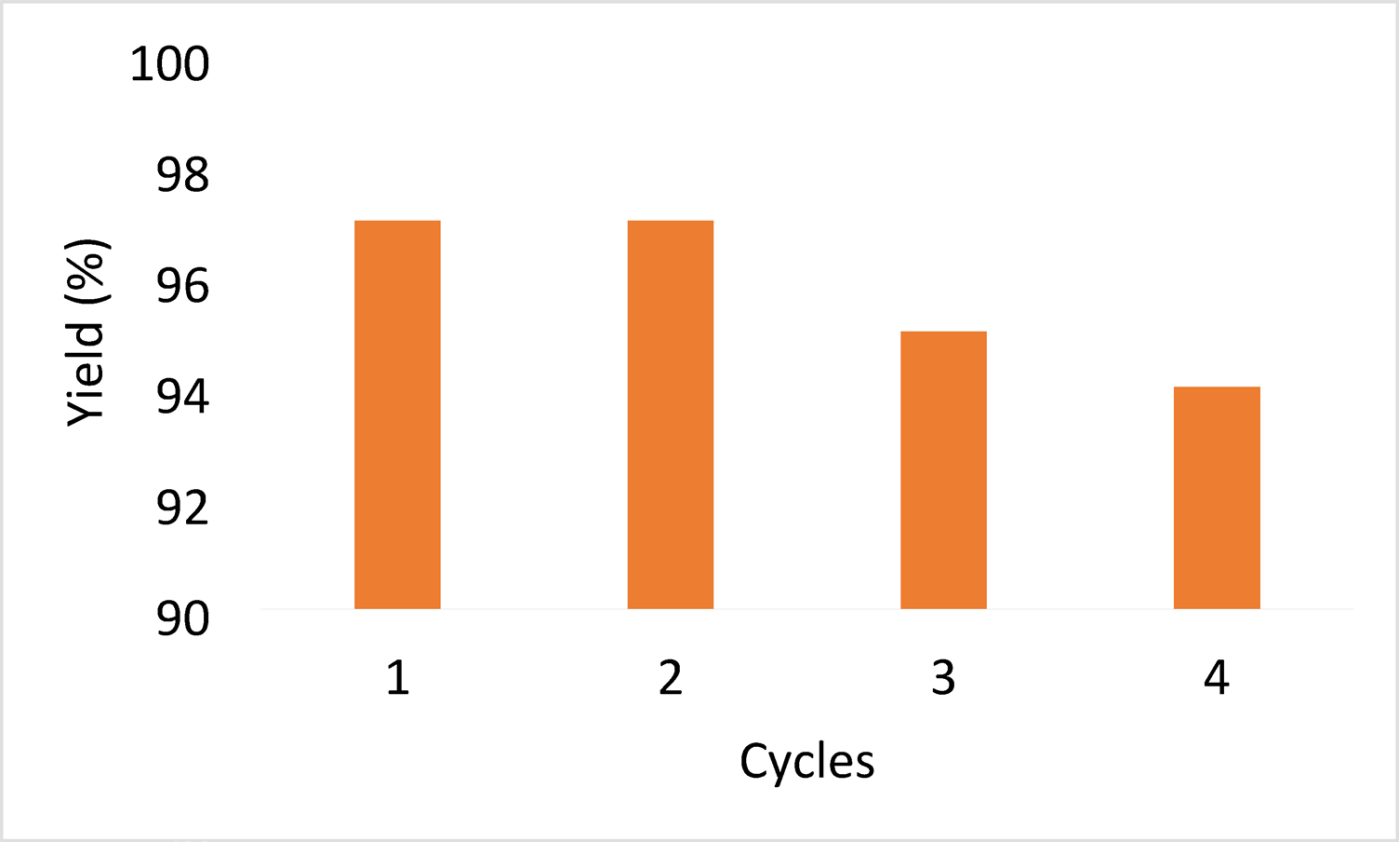
The effect of reusability of nanocrystalline ZnO/MgO on the synthesis of **4a**.

Moreover, the reusability of the developed heterogeneous catalyst was assessed using the model reaction comprising resorcinol (**2**), malononitrile (**3**), and benzaldehyde (**1a**) under optimum conditions (Table 1, entry 11). After grinding the reactants along with the catalyst for 8 min, DCM was added to dissolve the obtained product (**4a**). After filtration to recover the catalyst, washing it with ethanol removes any remaining organic impurities. Next, reusing the dried (at 80 °C under vacuum for 2 h) catalyst directly with fresh reactants, without additional purification, under ideal conditions. XRD, SEM, and FTIR analyses confirmed that the catalyst’s crystalline structure and chemical integrity were preserved (Figs. 10, 11 and 12). Figure 7 shows the data obtained over four cycles. The results in Fig. 7 undoubtedly demonstrate the outstanding reusability and stability of the nanocrystalline ZnO/MgO catalyst. Achieving yields of 97% in the first cycle and only a slight decrease to 94% by the fourth cycle indicates minimal loss in catalytic efficacy. This demonstrates that the catalyst maintains good activity over four consecutive cycles.

**Scheme 1:**
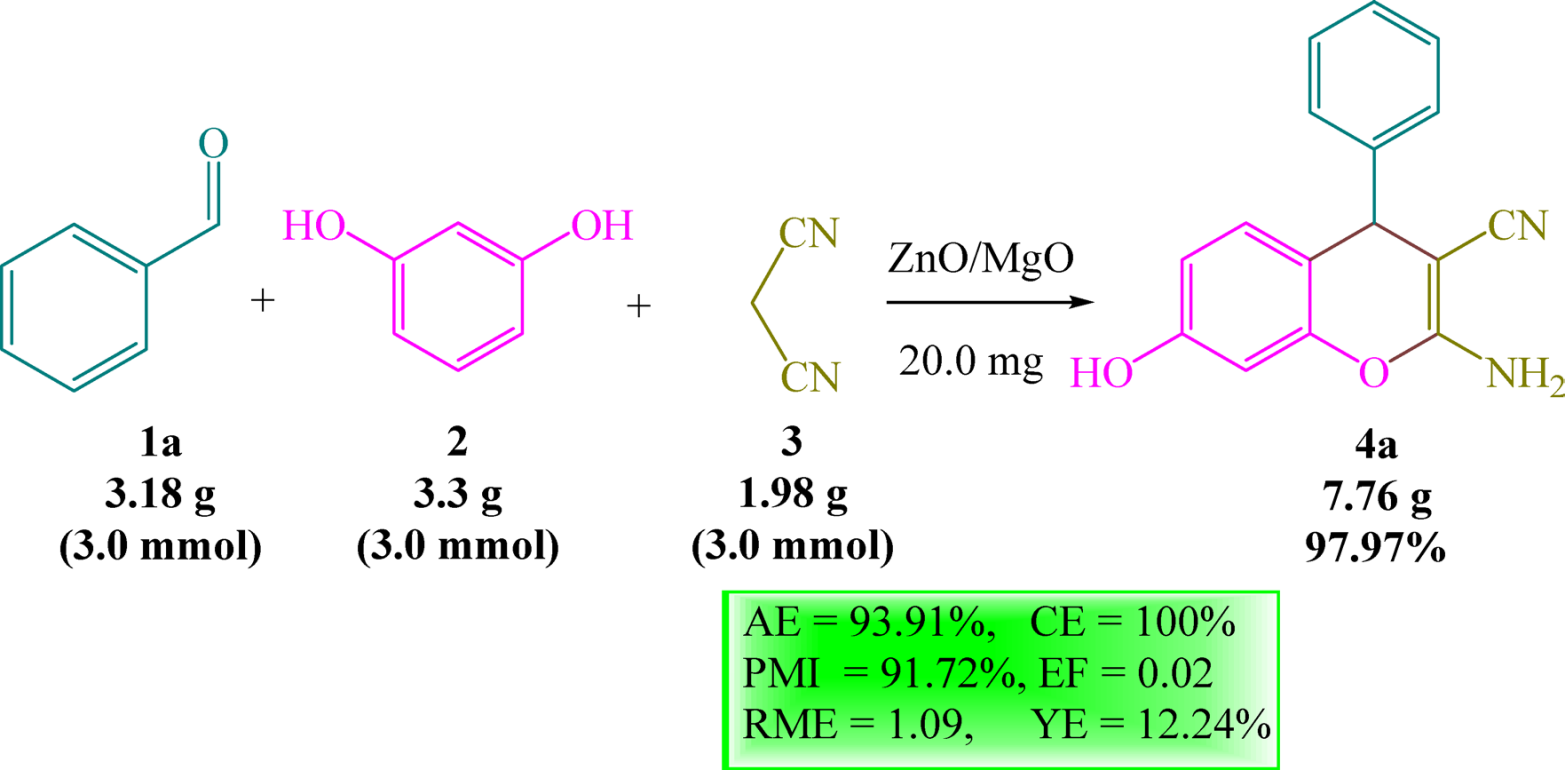
Scaled up the assembly of compound **4a**.

To determine the possibility of scaling up the developed procedure, the model reaction was performed on a gram scale (Scheme 1), yielding the desired substance (**4a**) with high yield (97.97%). Achieving excellent yields under gram-scale conditions demonstrates the robustness of the catalyst and supports its evaluation in future scale-up studies. Moreover, the greenness of the current protocol has been evaluated using a variety of eco-friendly parameters (Scheme 1)^33^. As shown in Scheme 1, high atom economy (AE = 93.91%) indicates that the majority of atoms from the reactants are retained in the end product, with minimal waste (high reaction efficiency). Moreover, achieving 100% carbon efficiency (CE) indicates that no undesirable carbon-containing by-products are produced throughout the process. Interestingly, the yield economy (YE) recorded a high score (12.24%), suggesting that the reaction is tailored to maximize the yield of the intended product relative to the starting reactants. Furthermore, the relatively high process mass intensity (PMI = 91.72%) and the low reaction mass efficiency (RME = 1.09) indicate that the reaction does not require excessive reactant use, offering an environmental benefit by minimizing material consumption. Finally, the environmental factor (EF) score of 0.02 indicates that the reaction has a limited negative environmental impact, which is an important principle of green chemistry. Thus, by maintaining high atom economy and carbon efficiency while minimizing waste, our method appears to align with several principles of green chemistry. While the chromene synthesis is carried out under solvent-free conditions, only a minimal amount of dichloromethane is used for catalyst separation and was recovered where possible. Thus, the overall procedure largely adheres to green chemistry principles by minimizing solvent use and promoting sustainability.

To assess the potential of the current study, the efficiency of the developed catalyst was compared with that of several others reported in the literature for preparing 2-amino-7-hydroxy-4-phenyl-4*H*-chromene-3-carbonitrile (**4a**), as shown in Table 3. The data listed clearly demonstrate that the ZnO/MgO NPs catalyst has considerable benefits over the other catalysts presented. The ZnO/MgO NPs accomplished a comparatively high yield of 97% with a very quick reaction time of only 8 min. In comparison, catalysts such as MgFe_2_O_4_ NPs require 12 min and yield 74%, underscoring the outstanding performance of ZnO/MgO NPs in improving yield and reducing reaction time. Although some catalysts, including MgO**–**NPs, Fe_3_O_4_**–**NPs, and Na_2_CO_3,_ offer somewhat improved yields (92–98%), they need significantly lengthier reaction periods (20**–**40 min). To evaluate sustainability, we also compared the Process Mass Intensity (PMI) and Environmental Factor (EF) of our protocol with representative chromene syntheses. Our ZnO/MgO-catalyzed, solvent-free method exhibits a high PMI (91.72%) and an exceptionally low EF (0.02), indicating minimal waste (Scheme 1). By comparison, the TEA-catalyzed solvent-free protocol (PMI 76.71%, EF 0.303)^34^, the Na₂CO₃-catalyzed solvent-free protocol (PMI 86.05%, EF 0.16)^36^, and the catalyst-free solvent-free protocol (PMI 87.21%, EF 0.13)^37^ generate significantly more waste, highlighting the superior environmental efficiency of our approach. Consequently, ZnO/MgO NPs act as highly effective catalysts, offering short reaction times, solvent-free conditions, and high yields, and demonstrating competitive performance compared with several reported catalytic systems.

**Table 3:** Comparative analysis of the present method vs. earlier reports.

No.	Catalyst	Solvent	Time (min)	Method/T (°C)	Yield (%)	Refs.
1.	TEA	–	3	MW (300 W)	82	^34^
2.	Triethanolamine	H_2_O	45	Reflux	92	^35^
3.	Na_2_CO_3_	**–**	40	Grinding/50	92	^36^
4.	**–**	**–**	20	CFL* irradiation (22 W)/rt	92	^37^
5.	MgFe_2_O_4_ NPs	EtOH	12	US/65	74	^38^
6.	Na_2_CO_3_	H_2_O: MeOH 95:5	600	25	72	^39^
7.	MgO**–**NPs	H_2_O	20	rt	98	^40^
8.	**–**	PEG-400	65	100	90	^41^
9.	Fe_3_O_4_**–**NPs	H_2_O	25	rt	98	^42^
10.	HAp**–**CoFe_2_O_4_	EtOH	15	MW (300 W)	88	^43^
11.	Fe_3_O_4_@SiO_2_/Schiff base of Cu(II)	H_2_O	40	rt	94	^44^
12.	Fe_3_O_4_**–**DOPA**–**Cu	EtOH	8	MW (100 W)/60	95	^45^
13.	ZnO/MgO NPs	**–**	8	rt	97	This work

*CFL = Compact fluorescent lamp.

**Scheme 2:**
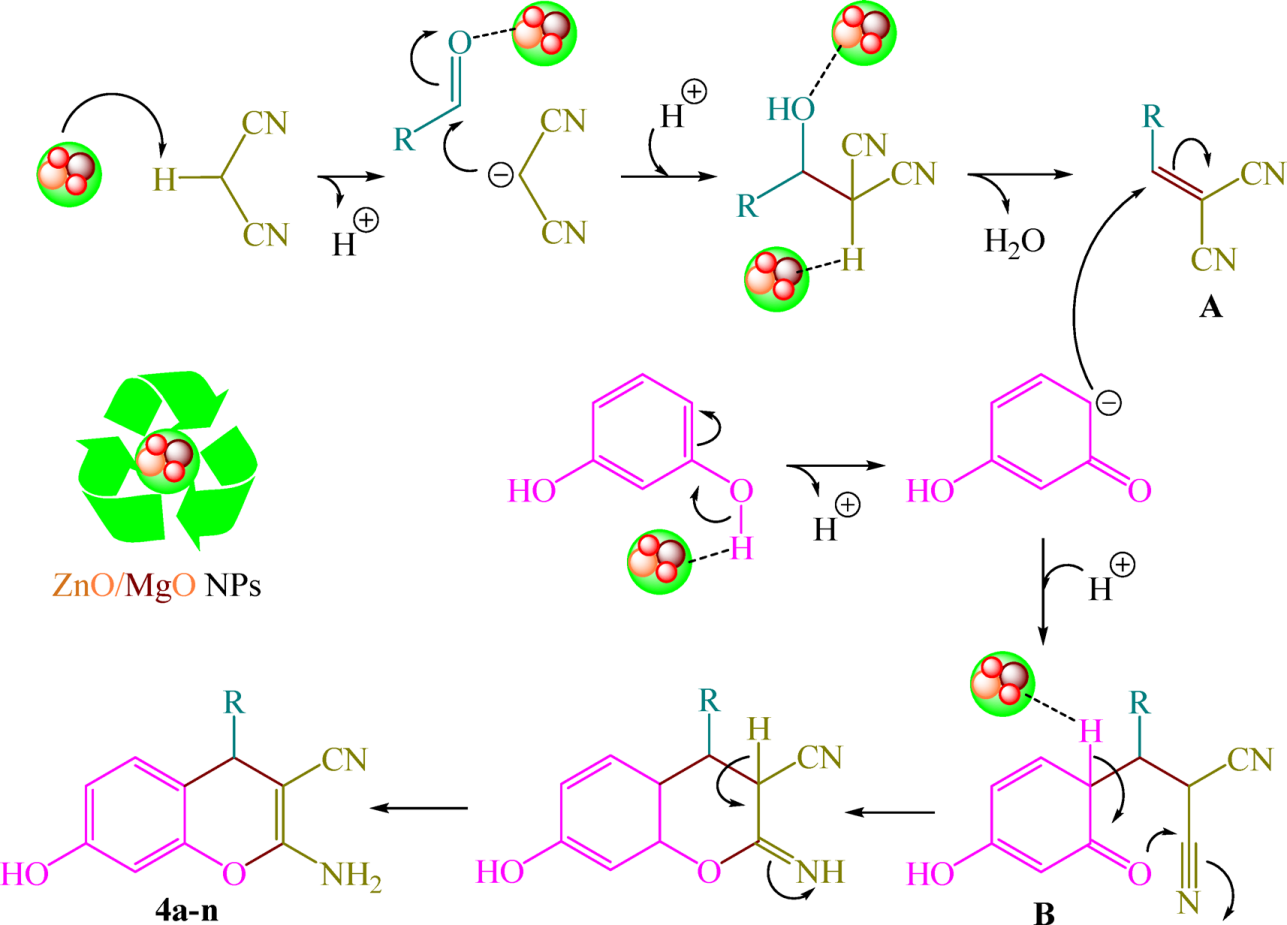
The proposed mechanistic pathway for the assembly of 2-amino-4*H*-chromenes (**4a–n**) catalyzed by ZnO/MgO NPs.

A proposed mechanism that accounts for the observed results is shown in Scheme 2. This pathway includes a typical sequence of Knoevenagel condensation, Michael addition, and cyclization, promoted by ZnO/MgO NPs (Scheme 2). As depicted, ZnO/MgO NPs initially activate the carbonyl group of aldehydes, decreasing the transition state’s energy, which assists in the nucleophilic attack of malononitrile. The reaction is predicted to proceed *via* Knoevenagel condensation to form intermediate **A**, followed by a Michael addition to generate intermediate **B**. This is followed by cyclization and tautomerization, ultimately leading to the formation of products **4a–n**.

### Photocatalytic activity

**Fig. 8:**
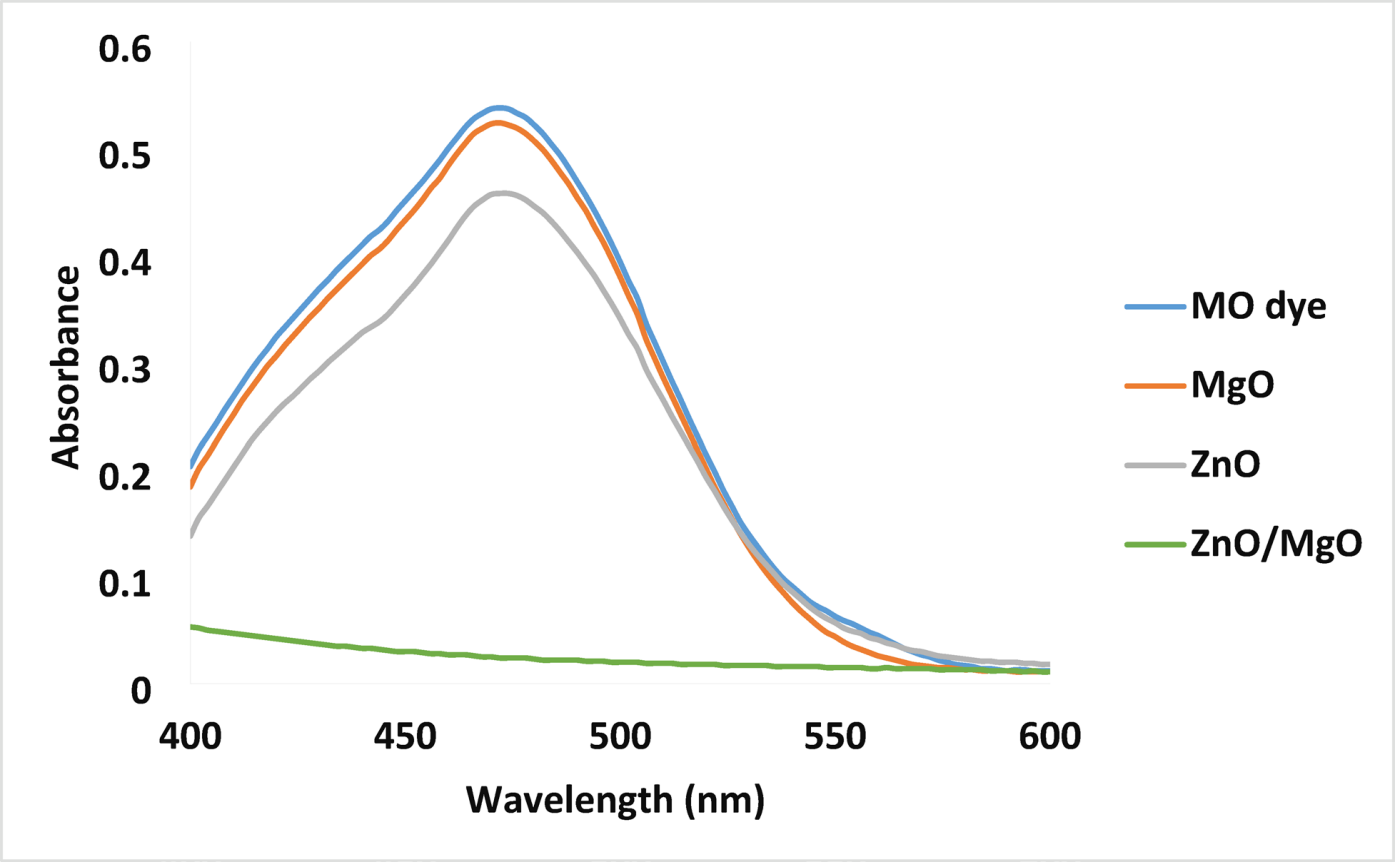
Absorbance spectra of MO dye after sunlight irradiation in the presence of MgO, ZnO, and ZnO/MgO as photocatalysts.

The photocatalytic performance of ZnO/MgO solid solution has been evaluated against the MO dye as a model of organic pollutants and compared with that of ZnO and MgO single oxides (Fig. 8).

The removal efficiency (%) is calculated using Eq. (1)^46^:

1 $${Removal}{\text{ }}\% = \frac{{A0 - At}}{{A0}} \times 100$$

where *At* is the absorbance of the MO after 30 min, and *A*_*0*_ is the initial 5.0 ppm MO absorbance (λ_max_ = 465 nm). The removal percentages in the presence of MgO, ZnO, and ZnO/MgO photocatalysts after 30 min were 3.64%, 18.18%, and 96.36%, respectively.

**Fig. 9:**
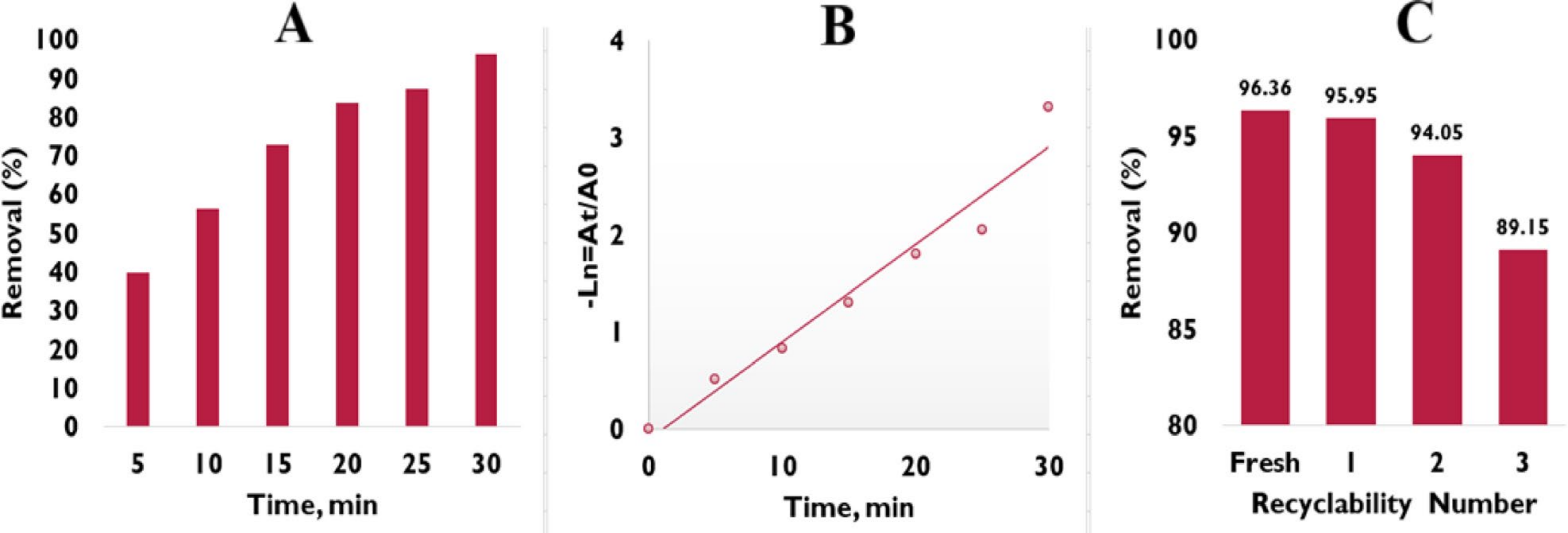
Effect of contact time on the degradation of MO dye using ZnO/MgO photocatalyst (**A**). Pseudo first order kinetics of MO degradation (**B**). Recyclability of ZnO/MgO (**C**).

Control experiments were performed to distinguish adsorption from photocatalytic degradation. Photolysis of methyl orange (pH = 7.6) under sunlight in the absence of ZnO/MgO showed negligible degradation. Adsorption in the dark led to a removal efficiency of 25.73% after 30 min, indicating some MO uptake on the catalyst surface. In contrast, under sunlight in the presence of ZnO/MgO (2.0 g/L), MO degradation reached 96.36% within 30 min. These results indicate that the observed MO removal is predominantly due to the photocatalytic activity of ZnO/MgO under natural solar light. Figure 9A displays the effect of the reaction period on the photocatalytic removal of MO. As shown, the removal efficiency of MO is 96.36% after 30 min of contact reaction time. The Langmuir-Hinshelwood model^47^, Eq. (2), has been used to determine the reaction kinetics of the photocatalytic removal of MO in the presence of ZnO/MgO under solar irradiation.

2 $${\text{ - Ln = At/A0 = kt}}$$

Where *A₀* is the initial concentration of MO, *At* is the concentration of MO after reaction time, *k* is the rate constant, and *t* is MO degradation time. The linear relationship, Fig. 9B, with calculated rate constant (k) of 0.1002 min^− 1^ and correlation coefficient (R² = 0.9549), confirms that the pseudo-first-order model is fitted for MO photocatalytic degradation under the investigated conditions. The higher photocatalytic activity of ZnO/MgO than that of individual ZnO and MgO may be referred to as the synergistic effect^48^. The recyclability of ZnO/MgO was tested three times under the same reaction conditions and showed removal efficiencies of 95.95%, 94.05%, and 89.15% (Fig. 8C). The reduction in the efficiency of the catalyst in the subsequent cycles can be explained by the changes that occur in the surface morphology of the catalyst; minute changes on the surface of the catalyst may result in a reduction in the number of accessible active sites, thus slightly reducing its efficiency.

To identify the optimal catalyst loading, three doses of ZnO/MgO (0.05 g, 0.1 g, and 0.15 g) were tested with 50.0 mL of 5.0 ppm MO solution for 30 min. The MO removal efficiency increased from 88.3 to 96.4% as the catalyst mass increased from 0.05 to 0.1 g, due to the greater number of active sites available for adsorption^49^. Increasing the catalyst dose to 0.15 g did not lead to further significant degradation, indicating that the optimal catalyst loading for effective MO removal is 0.1 g per 50 mL solution (2.0 g/L).

### Evaluation of ZnO/MgO stability after catalytic applications

**Fig. 10:**
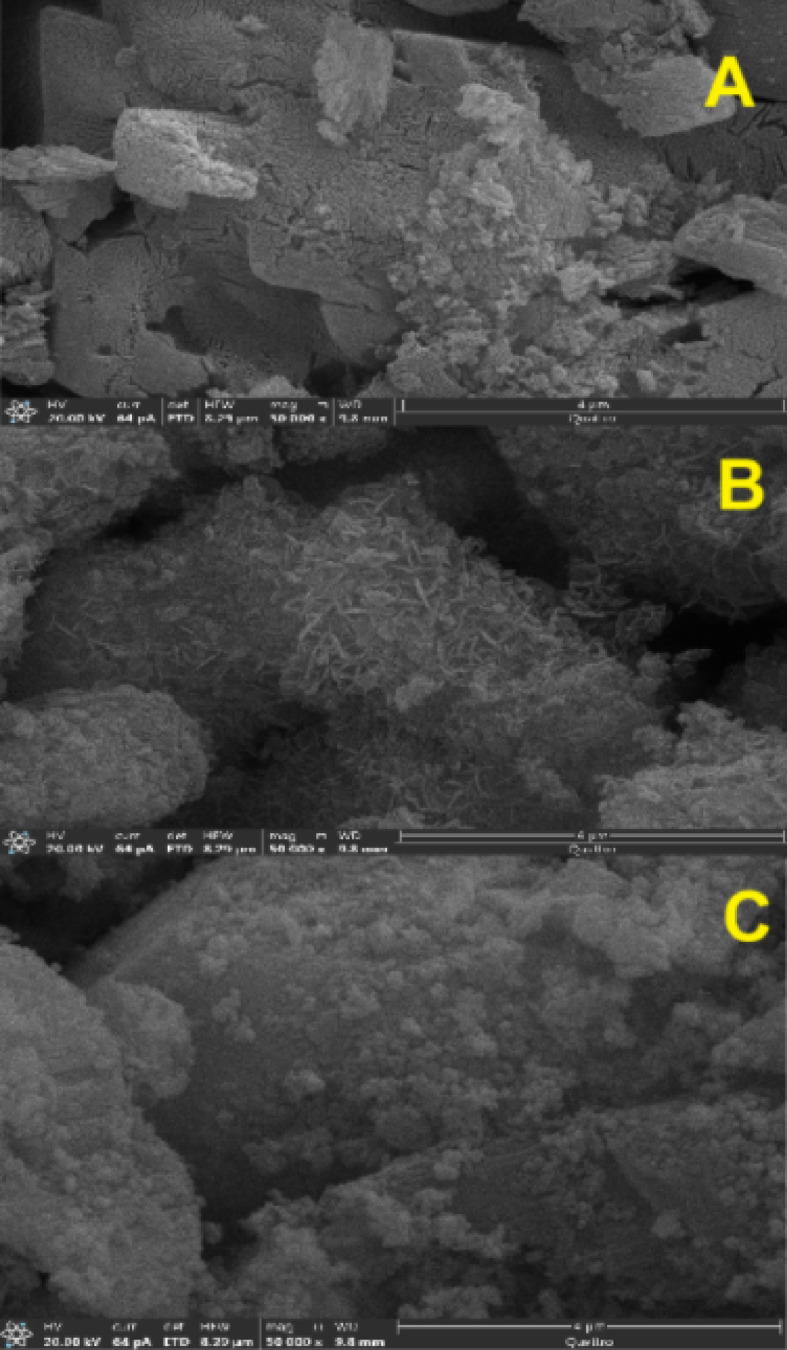
SEM images of ZnO/MgO before (**A**), after photocatalysis (**B**), and after catalytic reaction (**C**).

**Fig. 11:**
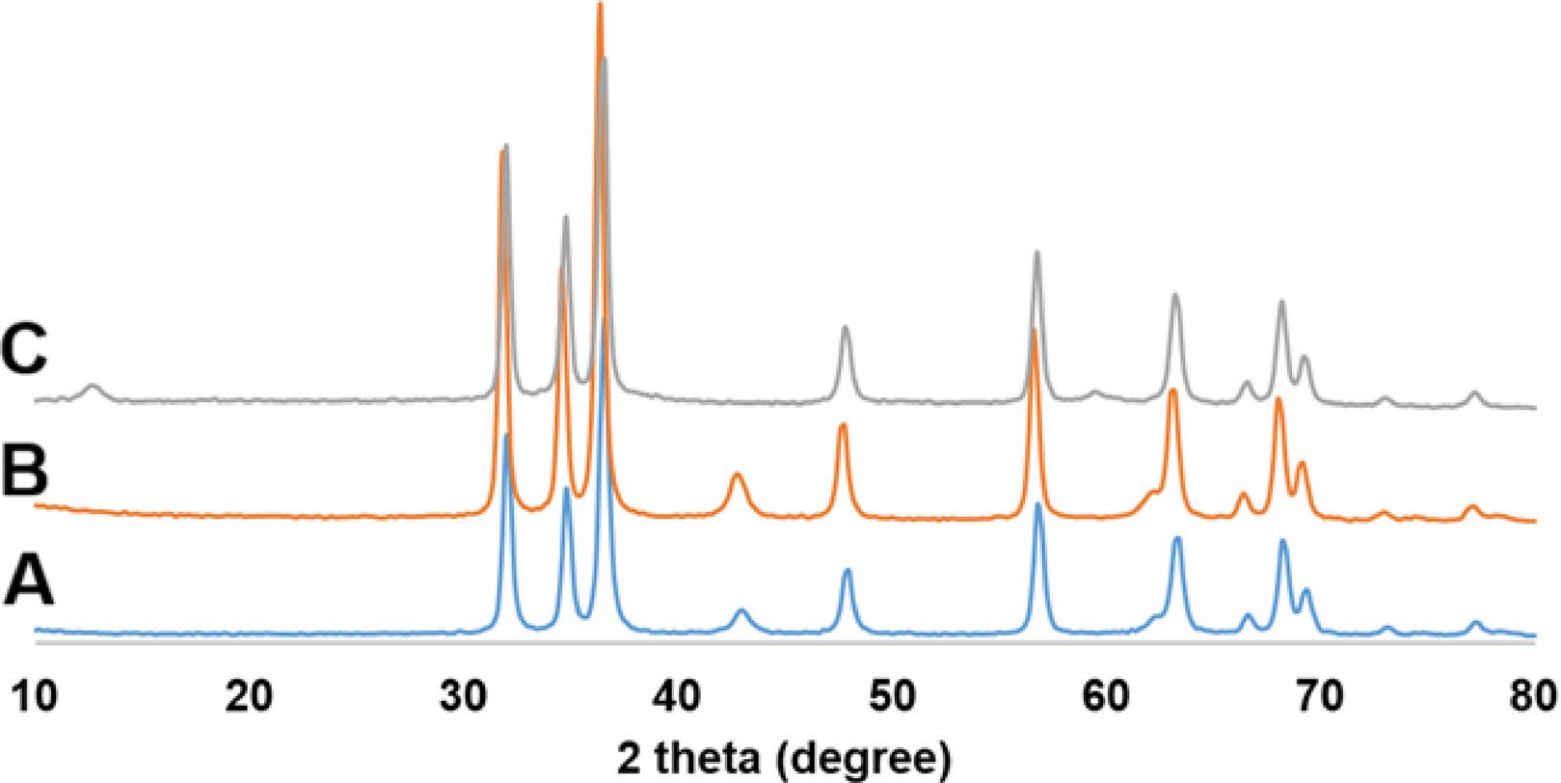
XRD patterns of ZnO/MgO before (**A**), after photocatalysis (**B**), and after catalytic organic reaction (**C**).

**Fig. 12:**
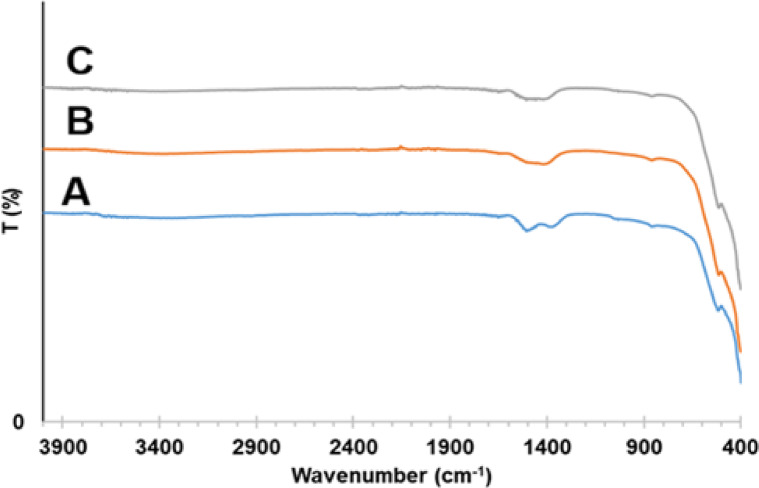
FTIR spectra of ZnO/MgO before (**A**), after photocatalysis (**B**), and after catalytic organic reaction (**C**).

The structural, crystalline, and compositional stability of the ZnO/MgO catalyst was systematically investigated by SEM, XRD, and FTIR spectroscopy (Figs. 10, 11 and 12). The measurements were conducted before (Figs. 10A, 11A, and 12B) and after (Figs. 9C, 10B, 11B, C, and 12B, C) its use in the photocatalytic degradation of methyl orange and in the catalytic organic reaction. Even as the SEM micrographs (Fig. 10) show distinct morphological transformation from well-defined crystalline in the pristine state (Fig. 10A) to a surface with more nanoflakes/needles upon photocatalytic dye degradation (Fig. 10B) and further on to granular and aggregated upon organic synthesis (Fig. 10C), the core properties are unchanged. Indeed, for all these three cases, the XRD patterns of the recovered catalyst (Fig. 11B, C) exhibit identical diffraction peaks, indicating that the bulk crystalline phase and hexagonal/cubic lattice integrity of ZnO/MgO have been preserved perfectly. This suggests that morphological changes as evidenced from SEM are surface-level changes without the interior of the material being disrupted. In parallel, FTIR spectra (Fig. 12), revealed no significant frequency shifts or appearance of new functional groups, supplementing the stability in ZnO/MgO bond linkages even under harsh conditions in photocatalytic or organic reactions. This synergy makes ZnO/MgO an excellent bifunctional catalyst. Therein, in the role of a photocatalyst, the ZnO/MgO heterojunction effectively hampers electron-hole recombination. Note that surface modification, as seen in image 10B, may be viewed as increasing the active surface area and, therefore, light-harvesting capability and leading to fast degradation of methyl orange. Next, as a heterogeneous catalyst in organic synthesis, the material offers stable basic sites/active sites. The aggregation, as noted in SEM image 10 C, may be considered as forming an active surface layer on the material on which the organic transformation takes place without any leaching or change in the catalyst’s fundamental chemical nature.

## Conclusion

A nanostructured composite (ZnO/MgO) was successively prepared *via* thermal decomposition of a mixed Zn/Mg oxalate precursor. Several tools (XRD, SEM, TGA, and FTIR) were employed to confirm its high purity, thermal stability, and crystalline structure (33 nm). When applied as a heterogeneous catalyst, ZnO/MgO enabled an efficient solvent-free Knoevenagel-Michael domino synthesis of a variety of 2-amino-4*H*-chromenes, providing high yields (91–99%) within short reaction times (8–12 min) at room temperature. The catalyst could be recovered and reused for up to four cycles with minimal loss of activity. In parallel, ZnO/MgO showed enhanced photocatalytic performance for the degradation of methyl orange under solar irradiation, achieving a removal efficiency of 96.36% within 30 min and outperforming the individual oxide components.

## Limitations and recommendations for future work

This study was limited to the degradation of a single anionic dye (MO). The catalyst’s performance with other dye types and its applicability in real or complex wastewater matrices, is currently being investigated in a separate study. Future work will also explore its applications in additional classes of organic reactions and under broader operational conditions, allowing a more comprehensive evaluation of its versatility and overall performance.

## Supplementary Information

Below is the link to the electronic supplementary material.

Supplementary Material 1

## Data Availability

All data generated or analyzed during this study are included in this published article.
