# Supplementary material for: Bifunctional recyclable ZnO/MgO nanocomposite: solvent-free synthesis of chromenes and efficient water remediation
Source: Sci Rep. 2026 May 7;16:14638. doi: 10.1038/s41598-026-43572-y (PMC13153398; doi:10.1038/s41598-026-43572-y)
Supplement: Supplementary file 1 — Supplementary Material 1 [file 41598_2026_43572_MOESM1_ESM.pdf]

Electronic Supporting Information

Bifunctional Recyclable ZnO/MgO Nanocomposite: Solvent-Free Synthesis of Chromenes and Efficient Water Remediation

Wael A. A. Arafa,[1]* AbdElAziz A. Nayl,[1] Ahmed H. Alanazi,[1] Ismail M. Ahmed,[1] Adel A. Abdelwahab,[1] Hamada Mohamed Ibrahim,[2] Stefan Bräse,[3]* Amr Mohammad Nassar[1]

[1]Department of Chemistry, College of Science, Jouf University, 72341, Sakaka, Al Jouf, Saudi Arabia

[2]Department of Chemistry, Faculty of Science, Fayoum University, 63514, Fayoum, Egypt

[3]Institute of Biological and Chemical Systems-Functional Molecular Systems (IBCS-FMS), Kaiserstrasse 12, 76131, Karlsruhe, Germany

Table of Contents

No.	Content	Page No.
1.	Instrumentation and materials	S1
2.	Synthesis of ZnO/MgO solid solution	S2
3.	General protocol for the preparation of 2-amino-4*H*-chromenes **4a–n**	S2
4.	Copies of NMR	S5
5.	Table S1: The calculated values of log (kX/kH) and σ	S18
6.	References	S18

Instrumentation and materials

A SHIMADZU TGA-51 was used for thermal gravimetric analysis (TGA). Scanning electron microscope (SEM) images were obtained using a Zeiss 1530 SEM. X-ray diffractions (XRD) were detected via XRD-7000 SHIMADZU with Kα copper radiation (1.5418 Å). A UV-Vis spectrophotometer (Cary 60 UV-Vis Spectrophotometer) was used to measure the absorption spectra. Fourier-Transform Infrared (FTIR) spectral data were measured on a Shimadzu IR-Tracer 100 spectrophotometer (Shimadzu Corporation, Kyoto, Japan). NMR spectra were acquired on a JEOL 600 MHz spectrometer (JEOL, Ltd., Peabody, MA, USA) utilizing DMSO-d6 as the solvent, with chemical shifts (δ) reported in ppm relative to tetramethylsilane (TMS) as an internal standard. Melting points were determined in open capillary tubes on an Electrothermal apparatus (temperature range 25–400 °C) and are uncorrected. All solvents and fine chemicals were purchased from Sigma-Aldrich (St. Louis, MO, USA) and used without further purification.

1.1. Synthesis of ZnO/MgO solid solution

The solid solution ZnO/MgO has been synthesized following the method described in our previous work [1], with the calcination time adjusted in order to improve phase homogeneity and ensure complete decomposition of the oxalate precursor. Thus, 1.26 g of oxalic acid dihydrate (10.0 mmol) was added to a mixture of aqueous solutions of zinc nitrate (1.49 g, 5.0 mmol) and magnesium nitrate (0.92 g, 5.0 mmol). The mixture was stirred for 1 hour at 70 °C. After that, the precipitate was filtered, washed several times with hot water, and left to dry at room temperature. The mixed oxalate sample was heated to 500 °C in a muffle for 3 hours to form a ZnO/MgO solid solution.

1.2. General protocol for the preparation of 2-amino-4*H*-chromenes 4a–n

A mixture of aldehydes (1.0 mmol), resorcinol (1.0 mmol), malononitrile (1.0 mmol), and MgO/ZnO (20.0 mg) was manually ground at room temperature using a standard laboratory mortar and pestle for 8 min. To ensure reproducibility, the grinding was performed under consistent conditions, applying comparable grinding time across all experiments. The progress of the reaction was monitored by TLC using a MeOH/DCM (1:9) solvent system, selected for its optimal separation of starting materials and products. Under these conditions, no significant side products were observed, and the reactions proceeded to complete conversion. Upon completion, the reaction mixture was extracted with dichloromethane (DCM) to remove the catalyst, and the pure products (4a–n) were obtained by evaporating the DCM under reduced pressure. The purity of all products was confirmed by ^{1}H NMR spectroscopy, melting point determination, and TLC, and all reported yields correspond to these isolated and purified products. The MgO/ZnO catalyst was separated from the reaction mixture by filtration, thoroughly washed with EtOH, dried at 80 °C under vacuum for 2 h, and reused in subsequent reactions without further activation, ensuring consistent catalytic activity across multiple cycles.

2-Amino-7-hydroxy-4-phenyl-4*H*-chromene-3-carbonitrile (4a)

Yellow solid; Yield: 97%; M.p. 230–232; IR, ν(cm^{-1}): 3397, 3309 (OH, NH), 2194 (C≡N), 1642,1597 (C=C); ^{1}H NMR (DMSO-*d6*, 600 MHz), *δ* (ppm): 4.81 (s, 1H, C*H*Ar), 6.12–6.18 (d, *J* = 3 Hz, 1H, Ar*H*), 6.58–6.63 (d, *J* = 9 Hz, 1H, Ar*H*), 6.79–6.84 (d, *J* = 9 Hz, 1H, Ar*H*), 6.93 (s, 2H, NH_2), 7.20–7.39 (m, 5H, Ar*H*), 9.86 (s, 1H, O*H*).

2-Amino-7-hydroxy-4-(4-methylphenyl)-4*H*-chromene-3-carbonitrile (4b)

Yellow solid; Yield: 96%; M.p. 187–189°C; IR, ν(cm^{-1}): 3381, 3279 (OH, NH), 2198 (C≡N), 1645,1583 (C=C); ^{1}H NMR (600 MHz, DMSO-*d6*), *δ* (ppm): 2.29 (s, 3H, CH_3), 4.69 (s, 1H, C*H*Ar), 6.11–6.14 (d, *J* = 9.3 Hz, 1H, Ar*H*), 6.68–6.73 (d, *J* = 9.3 Hz, 1H, Ar*H*), 6.82–6.87 (d, *J* = 10.5 Hz, 1H, Ar*H*), 7.08 (s, 2H, NH_2), 7.20–7.28 (d, *J* = 9.4 Hz, 2H, Ar*H*), 7.51–7.53 (d, *J* = 9.4 Hz, 2H, Ar*H*), 9.58 (s, 1H, O*H*).

2-Amino-7-hydroxy-4-(4-methoxyphenyl)-4*H*-chromene-3-carbonitrile (4c)

Yellow solid; Yield: 93%; M.p. 210–211 ◦C; IR, ν(cm^{-1}): 3369, 3291 (OH, NH), 2193 (C≡N), 1642,1580 (C=C); ^{1}H NMR (DMSO-*d6*, 500 MHz), *δ* (ppm): 3.70 (s, 3H, OCH_3), 4.54 (s, 1H, C*H*Ar), 6.16–6.19 (d, *J* = 8.8 Hz, 1H, Ar*H*), 6.41–6.44 (d, *J* = 8.8 Hz, 1H, Ar*H*), 6.76–6.77 (d, *J* = 8.5 Hz, 1H, Ar*H*), 6.96 (s, 2H, NH_2), 7.24–7.27 (d, *J* = 8.4 Hz, 2H, Ar*H*), 7.82–7.84 (d, *J* = 9.2 Hz, 2H, Ar*H*), 9.72 (s, 1H, O*H*).

2-Amino-7-hydroxy-4-(2-methoxyphenyl)-4*H*-chromene-3-carbonitrile (4d)

White solid; Yield: 91%; M.p. 222–224 ◦C; IR: *ν* (cm^{-1}) 3431, 3345–3324 (OH, NH), 2184 (C≡N), 1646, 1589 (C=C); ^{1}H NMR (600 MHz, DMSO-*d*$_6$), *δ* (ppm): 3.73 (s, 3H, OCH_3), 4.93 (s, 1H, C*H*Ar), 6.30–6.34 (d, *J* = 8.8 Hz, 1H, Ar*H*), 6.46–6.50 (d, *J* = 9.5 Hz, 1H, Ar*H*), 6.78–6.82 (d, *J* = 10, 1H, Ar*H*), 6.92-7.20 (m, 6H, Ar*H* +NH_2), 9.71 (s, 1H, O*H*).

2-Amino-7-hydroxy-4-(4-chlorophenyl)-4*H*-chromene-3-carbonitrile (4e)

Pale yellow solid; Yield: 97%; M.p. 161–163 °C; IR: ν (cm^{-1}): 3465, 3346, 3251 (OH, NH), 2190 (C≡N), 1645, 1519 (C=C); ^{1}H NMR (600 MHz, DMSO-*d*$_6$), *δ* (ppm): 4.62 (s, 1H, C*H*Ar), 6.36–6.37 (d, *J* = 3 Hz, 1H, Ar*H*), 6.44–6.49 (dd, *J* = 8.4, 3 Hz, 1H, Ar*H*), 6.73–6.75 (d, *J* = 8.4 Hz, 1H, Ar*H*), 6.86 (s, 2H, NH_2), 7.14–7.15 (d, *J* = 8.75 Hz, 2H, Ar*H*), 7.32–7.33 (d, *J* = 8.75 Hz, 2H, Ar*H*), 9.65 (br, 1H, O*H*).

2-Amino-7-hydroxy-4-(3-chlorophenyl)-4*H*-chromene-3-carbonitrile (4f)

Pale yellow crystals; Yield: 95%; M.p. 178–180 °C; IR: *ν* (cm^{-1}) 3427, 3338 (OH, NH), 2198 (C≡N), 1635, 1588 (C=C); ^{1}H NMR (600 MHz, DMSO-*d*$_6$): *δ* (ppm): 4.74(s, 1H, C*H*Ar), 6.36-6.37 (d, *J* = 3 Hz, 1H, Ar*H*), 6.46-6.49 (dd, *J* = 9, 3 Hz, 1H, Ar*H*), 6.60-6.61 (d, *J* = 8.5 Hz, 1H, Ar*H*), 6.97 (s, 2H, NH_2) 7.05–7.29 (m, 4H, Ar*H*), 9.86 (s, 1H, O*H*).

2-Amino-7-hydroxy-4-(2,4-dichlorophenyl)-4*H*-chromene-3-carbonitrile (4g)

Pale yellow crystals. Yield: 97%; M.p. 257–259 °C; IR, ν (cm^{-1}): 3512, 3461, 3308, 3255 (OH, NH), 2188 (C≡N), 1641, 1583 (C=C); ^{1}H NMR (600 MHz, DMSO-*d*$_6$), *δ* (ppm): 5.10 (s, 1H, C*H*Ar), 6.31–6.34 (d, *J* = 3 Hz, 1H, Ar*H*), 6.46–6.48 (d, *J* = 8.5 Hz, 1H, Ar*H*), 6.61–6.62 (d, *J* = 8.5 Hz, 1H, Ar*H*), 6.94 (s, 2H, NH_2), 7.11–7.14 (d, *J* = 8.4 Hz, 1H, Ar*H*), 7.32–7.34 (d, *J* = 8.4 Hz, 1H, Ar*H*), 7.51–7.53 (d, *J* = 2.0 Hz, 1H, Ar*H*), 9.76 (s, 1H, O*H*).

2-Amino-7-hydroxy-4-(4-bromophenyl)-4*H*-chromene-3-carbonitrile (4h)

Yellow solid; Yield: 96%; M.p. 223–224 °C; IR: ν (cm^{-1}): 3469, 3342, 3260 (OH, NH), 2189 (C≡N), 1661, 1524 (C=C); ^{1}H NMR (600 MHz, DMSO-d_6), δ (ppm): 4.79 (s, 1H, C*H*Ar), 6.33–6.34 (d, *J* = 3.5 Hz, 1H, Ar*H*), 6.44–6.45 (dd, *J* = 8.5, 3.5 Hz, 1H, Ar*H*), 6.68–6.70 (d, *J* = 8.5 Hz, 1H, Ar*H*), 6.91 (s, 2H, NH_2), 7.36–7.38 (d, *J* = 8.8 Hz, 2H, Ar*H*), 8.19–8.20 (d, *J* = 8.8 Hz, 2H, Ar*H*), 9.89 (br, 1H, O*H*).

2-Amino-7-hydroxy-4-(4-nitrophenyl)-4*H*-chromene-3-carbonitrile (4i)

Yellow crystals; Yield: 99%; M.p. 168–170 °C; IR, ν (cm^{-1}): 3469, 3321, 3217 (OH, NH), 2185 (C≡N), 1644, 1580 (C=C); ^{1}H NMR (600 MHz, DMSO-d_6), δ (ppm): 4.81 (s, 1H, C*H*Ar), 6.56–6.66 (m, 3H, Ar*H*), 6.92 (s, 2H, NH_2), 7.72–7.76 (d, *J* = 8.8 Hz, 2H, Ar*H*), 8.11–8.15 (d, *J* = 8.8 Hz, 2H, Ar*H*), 9.76 (s, 1H, O*H*).

2-Amino-7-hydroxy-4-(3-nitrophenyl)-4*H*-chromene-3-carbonitrile (4j)

Pale yellow crystals Yield: 97%; M.p. 171–172 °C; IR, ν (cm^{-1}): 3498, 3424, 3310 (OH, NH), 2189 (C≡N), 1661, 1584 (C=C); ^{1}H NMR (600 MHz, DMSO-d_6), δ (ppm): 4.80 (s, 1H, C*H*Ar), 6.17–6.20 (d, *J* = 3 Hz, 1H, Ar*H*), 6.56–6.59 (d, *J* = 8.5, 1H, Ar*H*), 6.74–6.77 (d, *J* = 8.5 Hz, 1H, Ar*H*), 6.96 (s, 2H, NH_2), 7.30–7.35 (m, 2H, Ar*H*), 7.84–7.87 (m, 2H, Ar*H*), 9.67 (s, 1H, O*H*).

2-Amino-7-hydroxy-4-(2-naphthyl)-4*H*-chromene-3-carbonitrile (4k)

Yellow crystals; Yield: 94%; M.p. 229–230 °C; IR: *ν* (cm^{-1}) 3421, 3337 (OH, NH), 2187 (C≡N), 1659, 1581 (C=C); ^{1}H NMR (600 MHz, DMSO-d$_6$): δ (ppm): 4.71 (s, 1H, C*H*Ar), 6.38–6.43 (d, *J* = 3, 1H, Ar*H*), 6.47–6.50 (d, *J* = 8, 1H, Ar*H*), 6.72–6.74 (d, *J* = 8, 1H, Ar*H*), 6.91 (s, 2H, NH_2), 7.20–7.81 (m, 7H, Ar*H*), 9.74 (s, 1H, O*H*).

2-Amino-7-hydroxy-4-(2-furyl)-4*H*-chromene-3-carbonitrile (4l)

Yellow crystals; Yield: 97%; M.p. 208–210 °C; IR: *ν* (cm^{-1}) 3481, 3424 (OH, NH), 2194 (C≡N), 1650, 1589 (C=C); ^{1}H NMR (600 MHz, DMSO-d_6): δ (ppm): 4.76 (s, 1H, C*H*Ar), 6.13–6.15 (d, *J* = 3, 1H, Ar*H*), 6.32–6.34 (d, *J* = 8, 1H, Ar*H*), 6.53–6.56 (d, *J* = 8, 1H, Ar*H*), 6.96 (s, 2H, NH_2), 7.31–7.45 (m, 3H, *H*-furyl), 9.77 (s, 1H, O*H*).

2-Amino-7-hydroxy-4-(ethyl)-4*H*-chromene-3-carbonitrile (4m)

Orange solid; Yield: 92%; M.p. 169–170 °C; IR: *ν* (cm^{-1}) 3467, 3340 (OH, NH_2), 2197 (C≡N), 1655, 1626 (C=C); ^{1}H NMR (600 MHz, DMSO-d_6), δ (ppm): 0.629–0.686 (t, *J* = 6 Hz, 3H, CH_3), 1.57–1.67 (qd, *J* = 6, *J* = 8 Hz, 2H, CH_2), 3.37–3.42 (t, *J* = 8 Hz, 1H, *H*-4), 6.31–6.34 (d, *J* = 2 Hz, 1H, Ar*H*), 6.51–6.53 (d, *J* = 2 Hz, 1H, Ar*H*), 6.74 (s, 2H, NH_2), 6.98–7.00 (d, 1H, *J* = 2 Hz, Ar*H*), 9.64 (s, 1H, O*H*).

1,4-Bis(2-amino-3-cyano-7-hydroxy-4*H*-chromen-4-yl)-benzene (4n)

Orange solid; Yield: 99%; M.p. >300 °C; IR: ν (cm^{-1}) 3420, 3324 (OH, NH), 2197 (C≡N), 1636, 1581 (C=C); ^{1}H NMR (600 MHz, DMSO-*d6*), δ (ppm): 4.65 (s, 2H, C*H*Ar), 6.31–6.32 (d, *J* = 3 Hz, 2H, Ar*H*), 6.48–6.50 (dd, *J* = 3 Hz, 2H, *J* = 8.8 Hz, Ar*H*), 6.77–6.78 (d, *J* = 8.8 Hz, 2H, Ar*H*), 6.91 (s, 4H, NH_2), 7.22 (s, 4H, Ar*H*), 9.90 (br, 2H, O*H*).

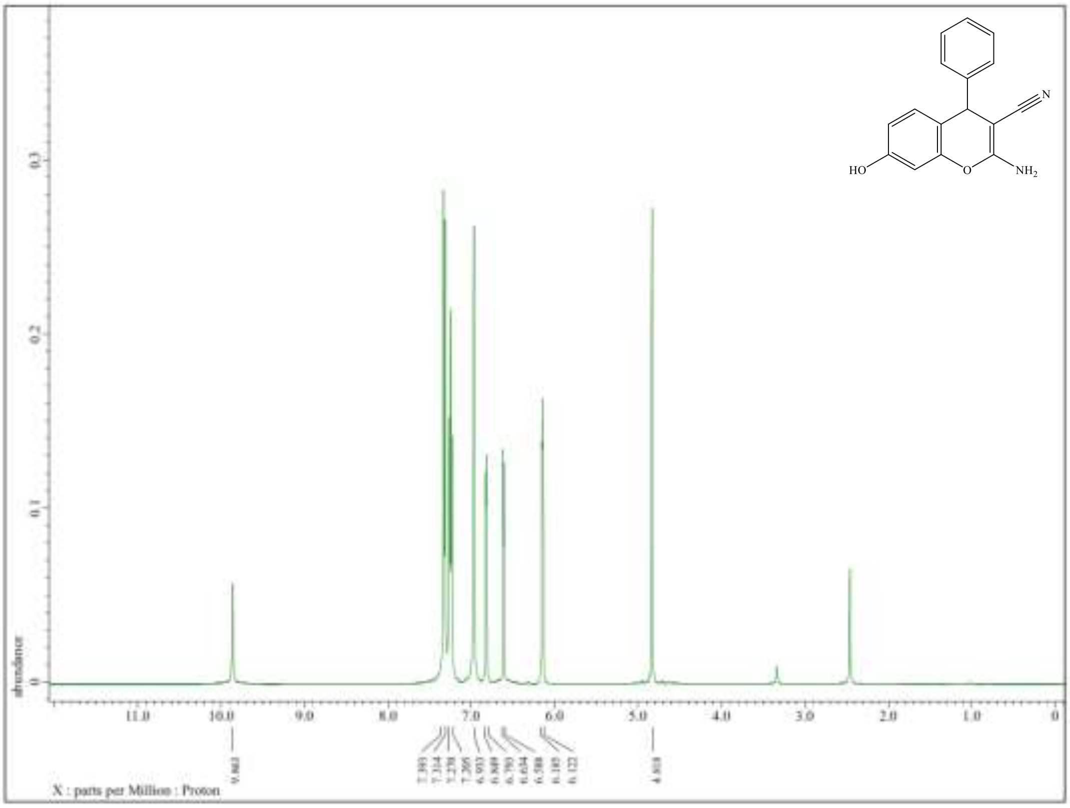

Fig. S1. ^{1}H NMR (DMSO-*d6*) of (**4a**)

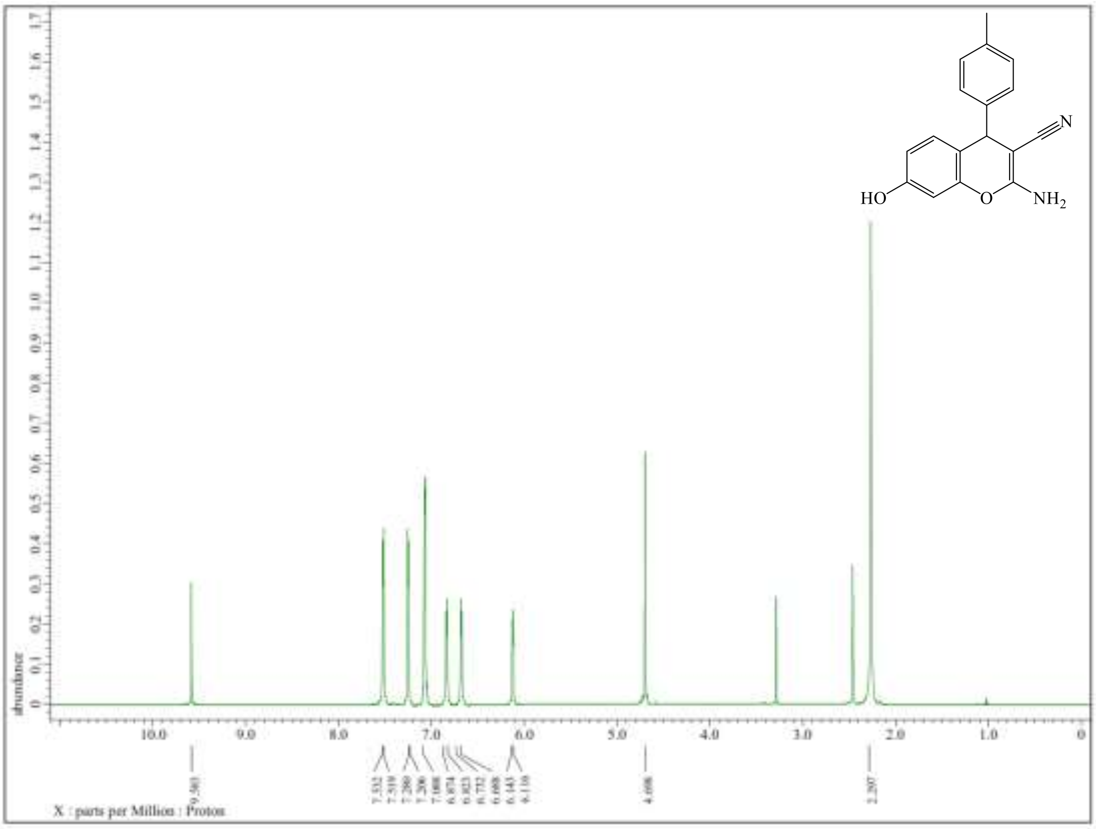

Fig. S2. ^{1}H NMR (DMSO-*d6*) of (**4b**)

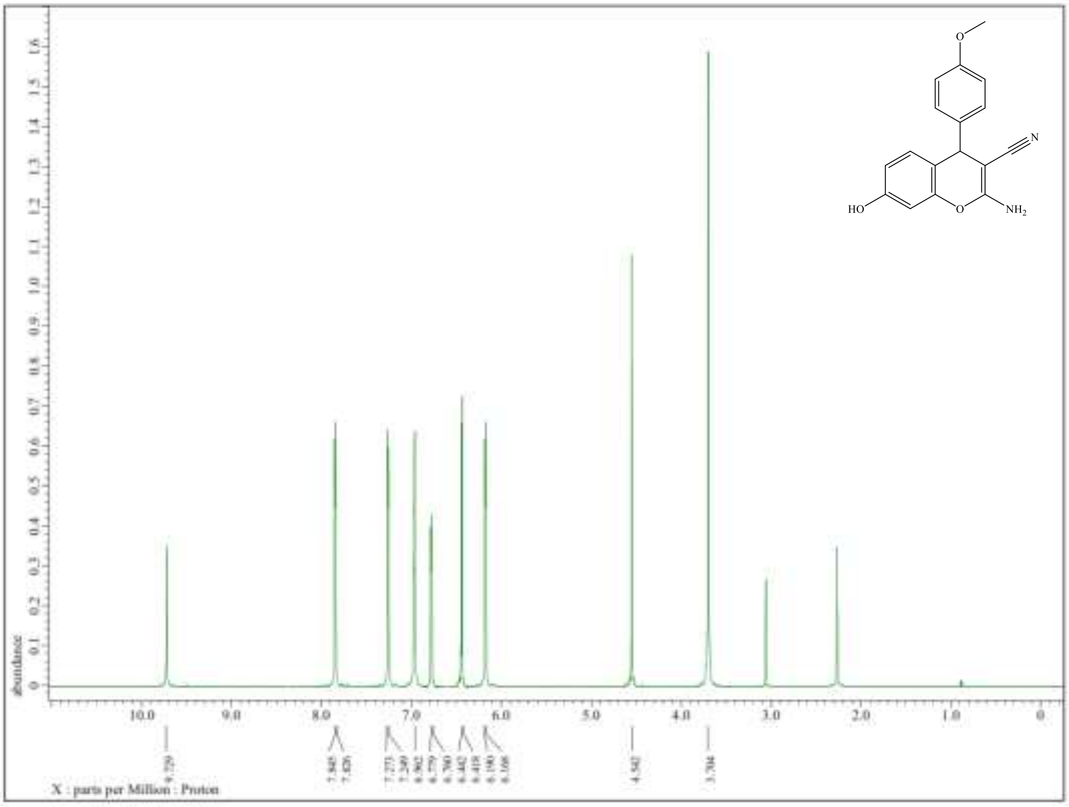

Fig. S3. ^{1}H NMR (DMSO-*d6*) of (**4c**)

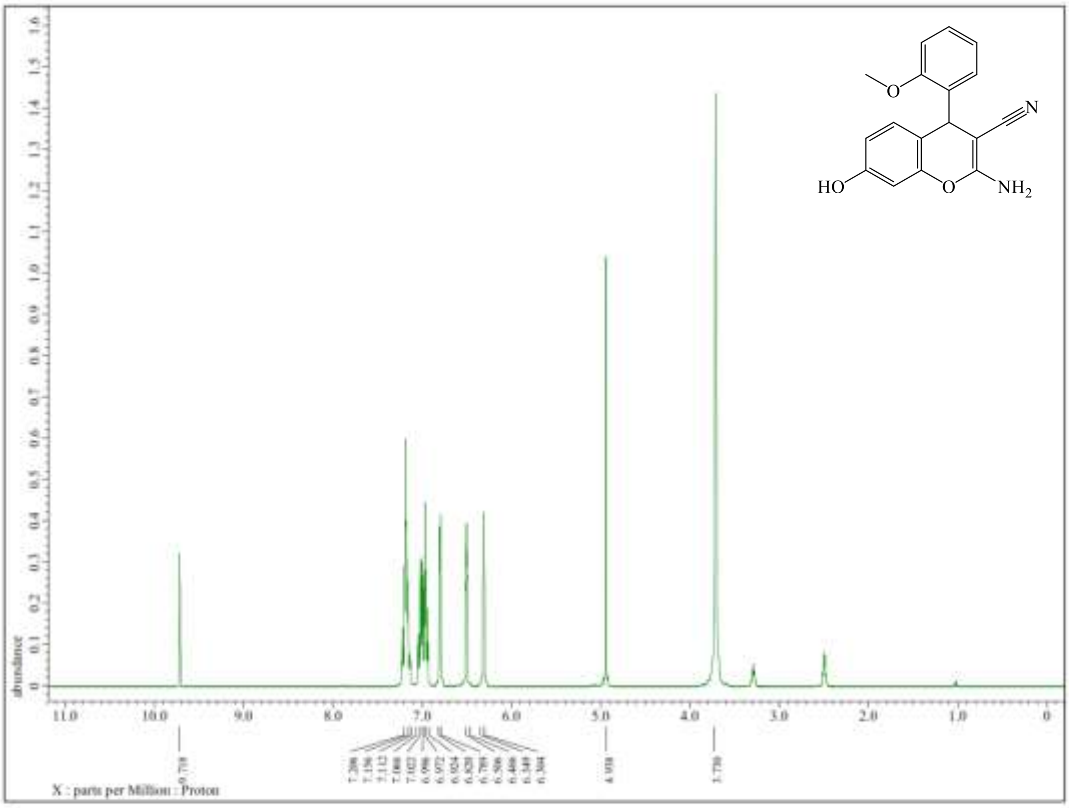

Fig. S4. ^{1}H NMR (DMSO-*d6*) of (**4d**)

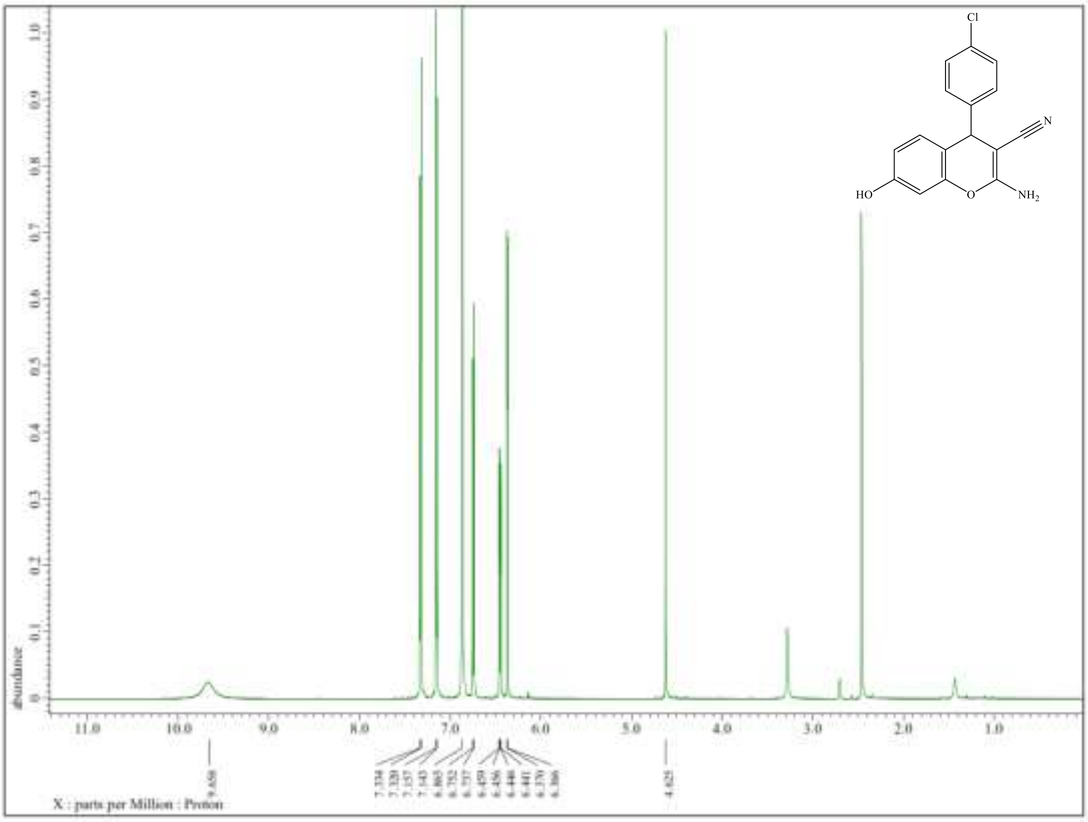

Fig. S5. ^{1}H NMR (DMSO-*d6*) of (**4e**)

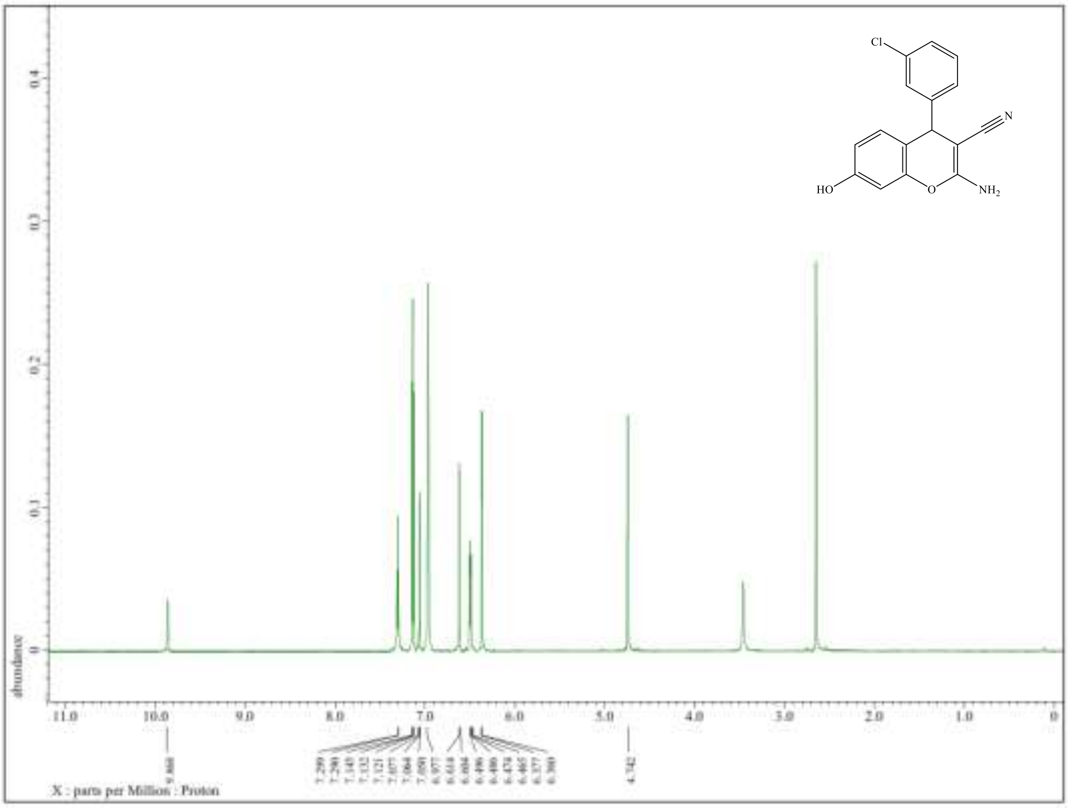

Fig. S6. ^{1}H NMR (DMSO-*d6*) of (**4f**)

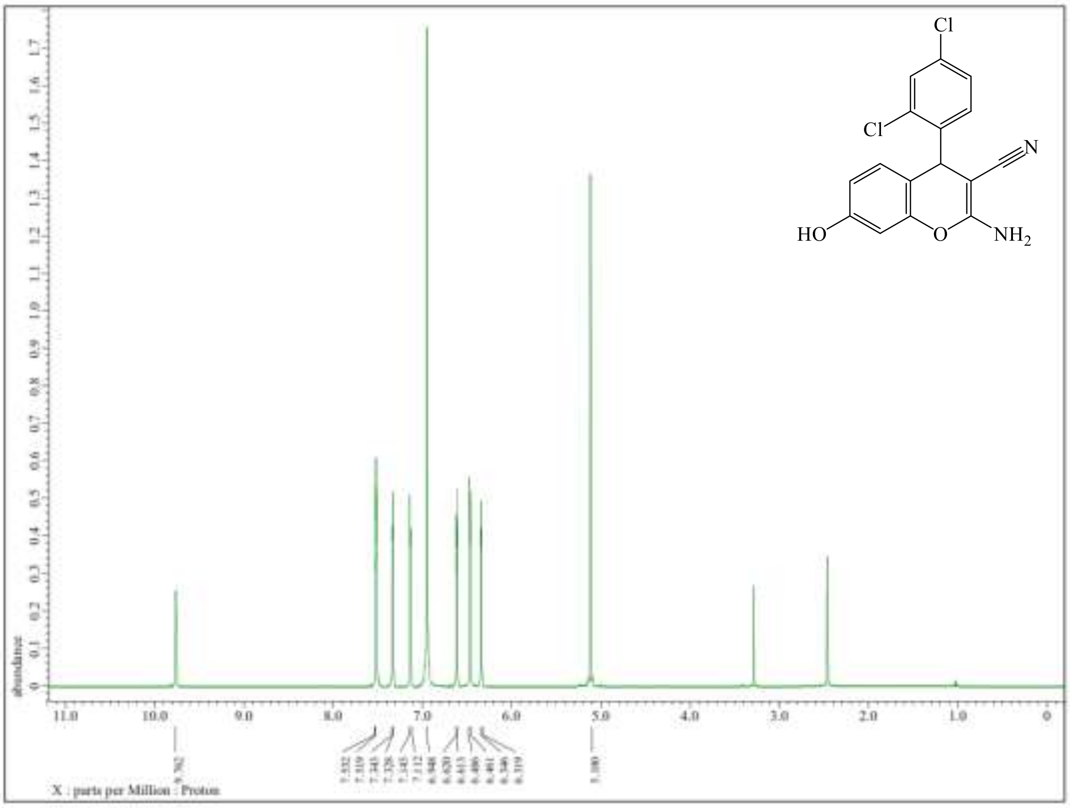

Fig. S7. ^{1}H NMR (DMSO-*d6*) of (**4g**)

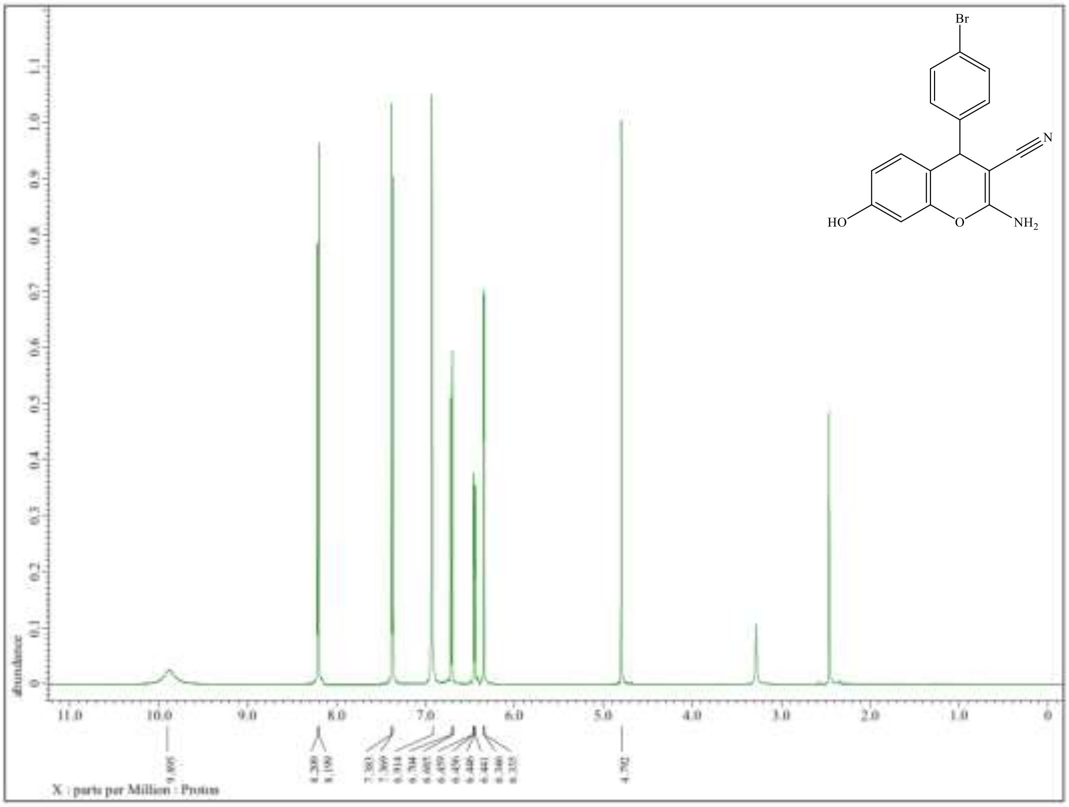

Fig. S8. ^{1}H NMR (DMSO-*d6*) of (**4h**)

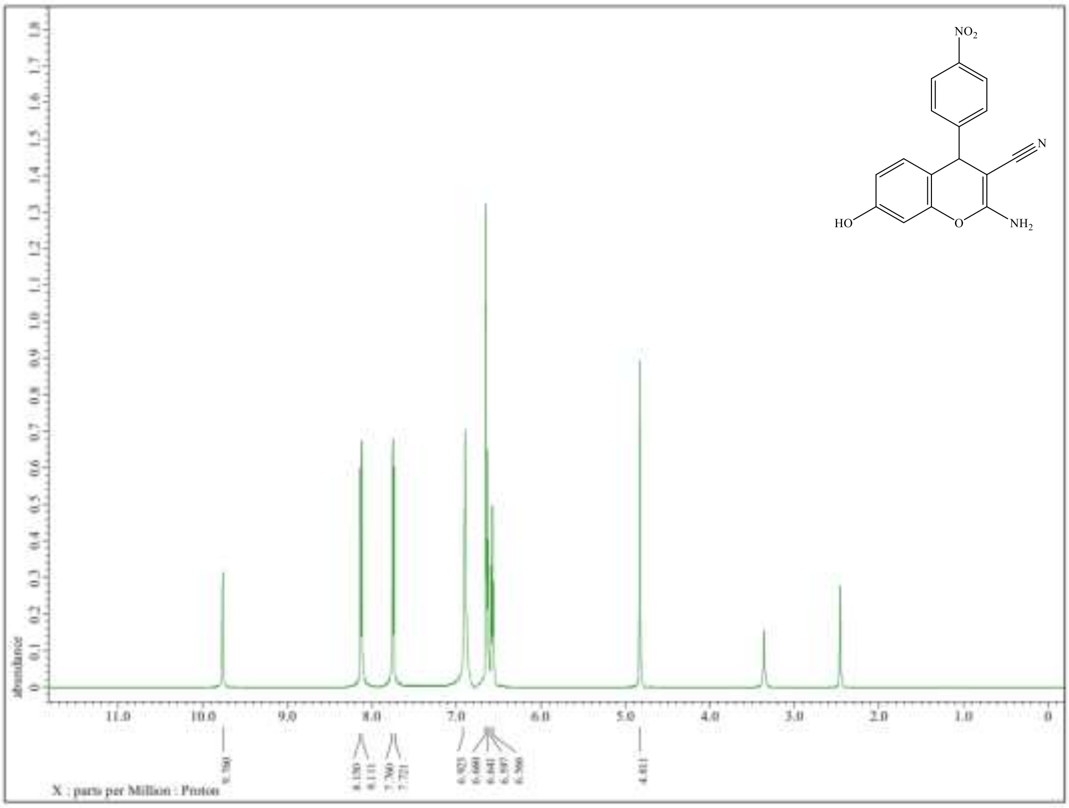

Fig. S9. ^{1}H NMR (DMSO-*d6*) of (**4i**)

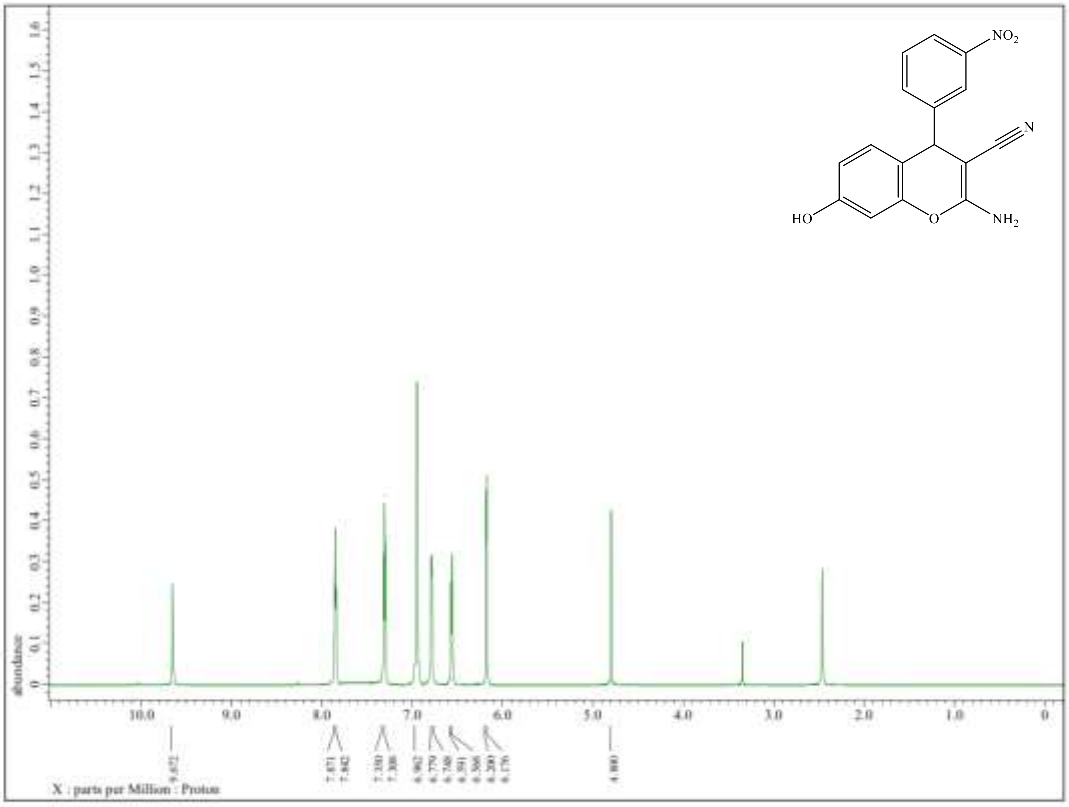

Fig. S10. ^{1}H NMR (DMSO-*d6*) of (**4j**)

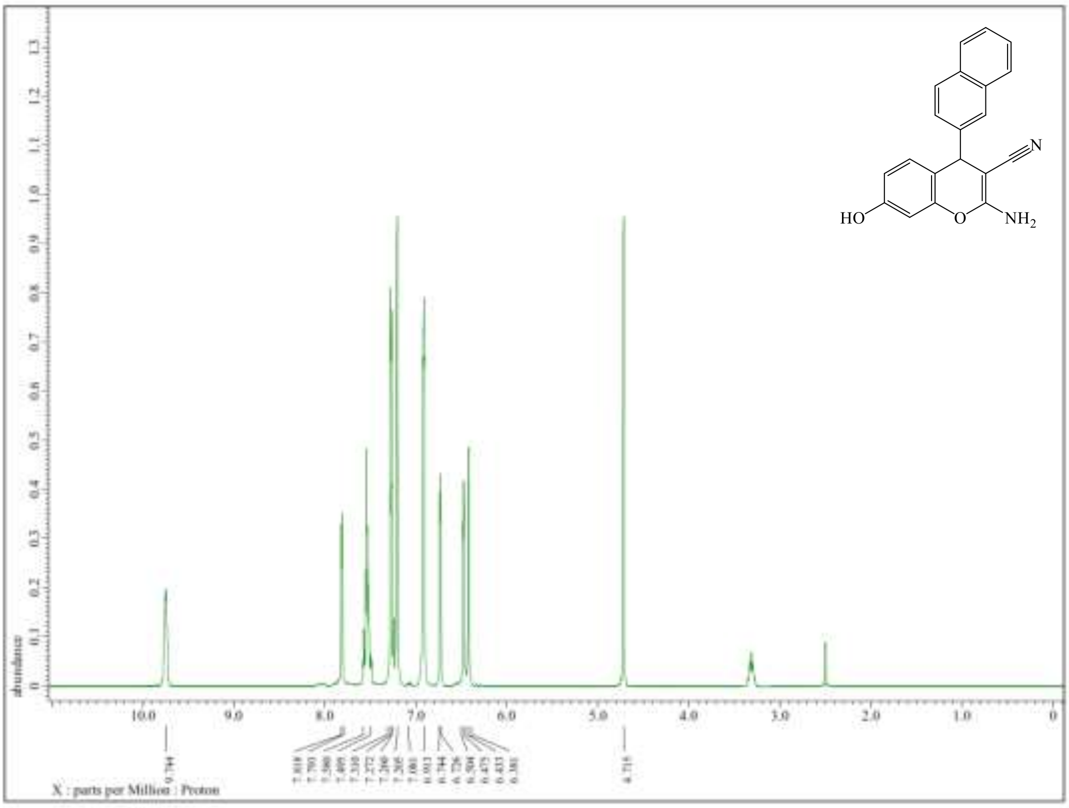

Fig. S11. ^{1}H NMR (DMSO-*d6*) of (**4k**)

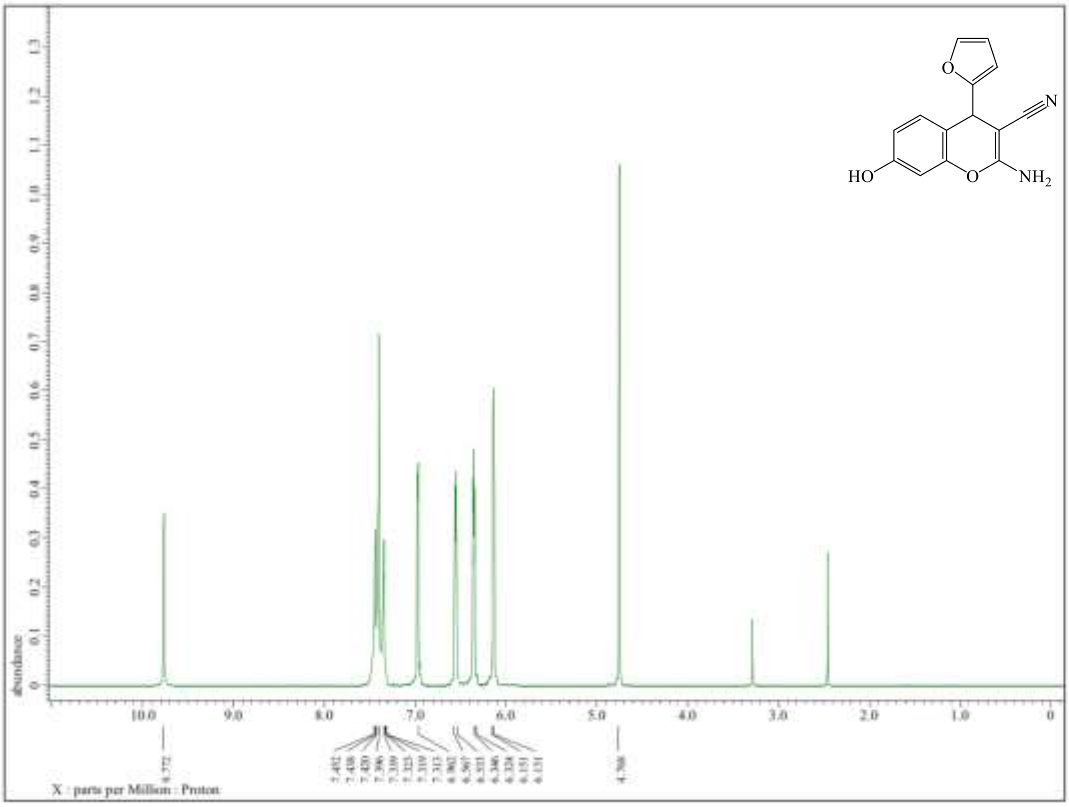

Fig. S12. ^{1}H NMR (DMSO-*d6*) of (**4l**)

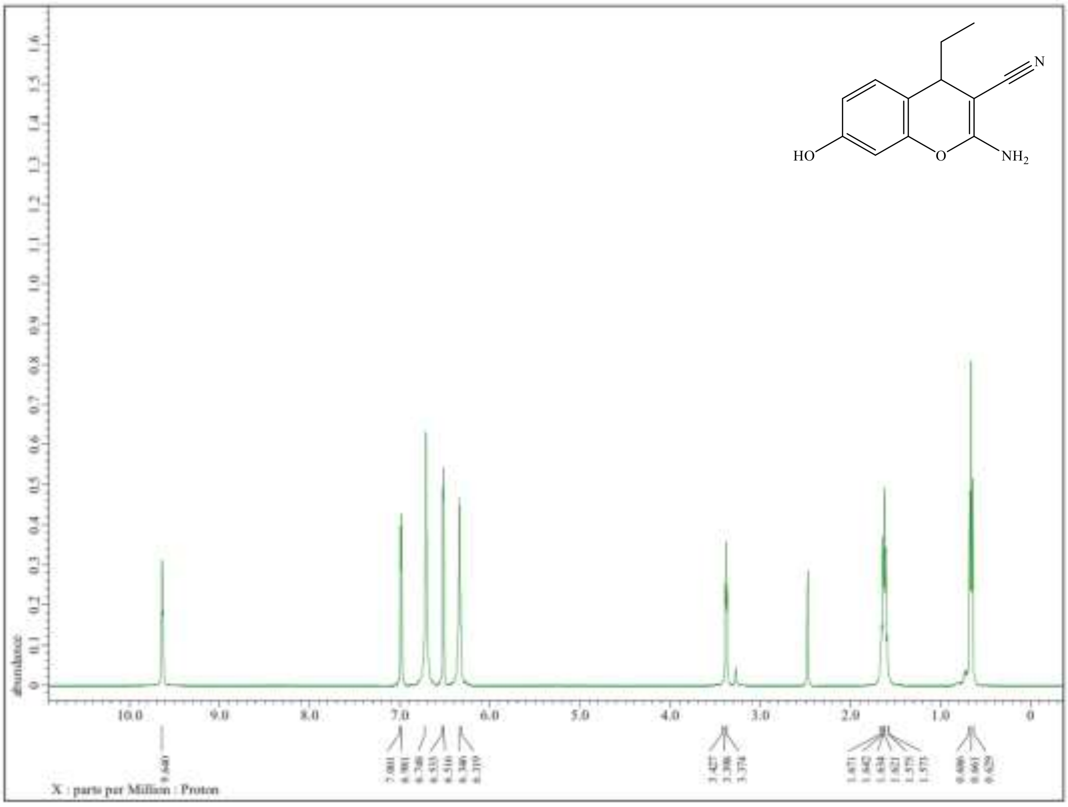

Fig. S13. ^{1}H NMR (DMSO-*d6*) of (**4m**)

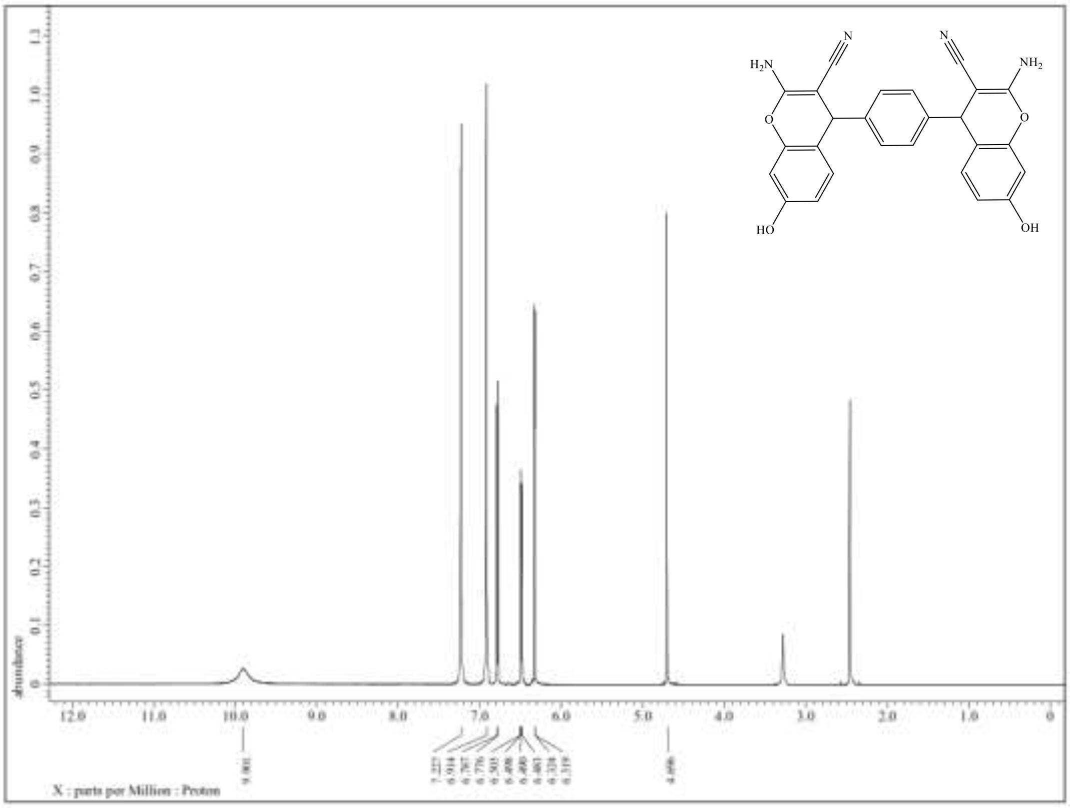

Fig. S14. ^1H NMR (DMSO-*d6*) of (**4n**)

Table S1. The calculated values of log (kX/kH) and σ.

Entry	R	Product	Time (min)	Yield (%)	σ	log (kX/kH)
1.	C_6H_5	**4a**	8	97	0	0
2.	4-MeC_6H_4	**4b**	8	96	-0.17	-0.005
3.	4-$MeOC_6H_4$	**4c**	8	93	-0.27	-0.018
4.	2-$MeOC_6H_4$	**4d**	10	91	-	-
5.	4-ClC_6H_4	**4e**	8	97	0.23	0
6.	3-ClC_6H_4	**4f**	8	95	0.37	-0.009
7.	2,4-$Cl_2C_6H_3$	**4g**	8	97	-	-
8.	4-BrC_6H_4	**4h**	10	96	0.23	-0.101
9.	4-$NO_2C_6H_4$	**4i**	8	99	0.78	0.009
10.	3-$NO_2C_6H_4$	**4j**	8	97	0.71	0
11.	2-Naphthyl	**4k**	8	94	-	-
12.	2-Furyl	**4l**	8	97	-	-
13.	Ethyl	**4m**	12	92	-	-
14.	$OHCC_6H_4$	**4n**	10	99	-	-

References

1. Dhafer CEB, Nassar AM, Alanazi AH, Alotaibi NF, Ali HM, Hasaneen MF, Moustafa SMN. Synthesis, Structure, Thermal, and Optical Analysis of Ag@MgO/ZnO: A Promising Nanocomposite for Antimicrobial and Antioxidant Applications. *ChemistrySelect*. 2025;10(38):e03173. https://doi.org/10.1002/slct.202503173
